# Inhibition of Connexin 36 attenuates HMGB1‐mediated depressive‐like behaviors induced by chronic unpredictable mild stress

**DOI:** 10.1002/brb3.2470

**Published:** 2022-01-28

**Authors:** Qian Jiang, Chao‐Ran Li, Wen‐Feng Zeng, Hui‐Jing Xu, Jia‐Mei Li, Ting Zhang, Guang‐Hui Deng, Yun‐Xia Wang

**Affiliations:** ^1^ Department of Psychiatry Faculty of Psychology Second Military Medical University Shanghai P. R. China; ^2^ Department of Nautical Psychology Faculty of Psychology Second Military Medical University Shanghai P. R. China; ^3^ Department of Stress Medicine Faculty of Psychology Second Military Medical University Shanghai P. R. China

**Keywords:** chronic unpredictable mild stress, Connexin 36, depressive disorder, hippocampal neurons, HMGB1

## Abstract

**Background:**

High mobility group box 1 (HMGB1) released by neurons and microglia was demonstrated to be an important mediator in depressive‐like behaviors induced by chronic unpredictable mild stress (CUMS), which could lead to the imbalance of two different metabolic approaches in kynurenine pathway (KP), thus enhancing glutamate transmission and exacerbating depressive‐like behaviors. Evidence showed that HMGB1 signaling might be regulated by Connexin (Cx) 36 in inflammatory diseases of central nervous system (CNS). Our study aimed to further explore the role of Cx36 in depressive‐like behaviors and its relationship with HMGB1.

**Methods:**

After 4‐week chronic stress, behavioral tests were conducted to evaluate depressive‐like behaviors, including sucrose preference test (SPT), tail suspension test (TST), forced swimming test (FST), and open field test (OFT). Western blot analysis and immunofluorescence staining were used to observe the expression and location of Cx36. Enzyme‐linked immunosorbent assay (ELISA) was adopted to detect the concentrations of inflammatory cytokines. And the excitability and inward currents of hippocampal neurons were recorded by whole‐cell patch clamping.

**Results:**

The expression of Cx36 was significantly increased in hippocampal neurons of mice exposed to CUMS, while treatment with glycyrrhizinic acid (GZA) or quinine could both down‐regulate Cx36 and alleviate depressive‐like behaviors. The proinflammatory cytokines like HMGB1, tumor necrosis factor alpha (TNF‐α), and interleukin‐1β (IL‐1β) were all elevated by CUMS, and application of GZA and quinine could decrease them. In addition, the enhanced excitability and inward currents of hippocampal neurons induced by lipopolysaccharide (LPS) could be reduced by either GZA or quinine.

**Conclusions:**

Inhibition of Cx36 in hippocampal neurons might attenuates HMGB1‐mediated depressive‐like behaviors induced by CUMS through down‐regulation of the proinflammatory cytokines and reduction of the excitability and intracellular ion overload.

## Graphical abstract

Inhibition of Cx36 in hippocampal neurons might attenuate HMGB1‐mediated depressive‐like behaviors induced by CUMS through down‐regulation of the proinflammatory cytokines and reduction of the excitability and intracellular ion overload.

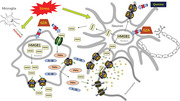

## 1 INTRODUCTION

Depressive disorder is a common mental disease characterized by depressed mood, anhedonia, and loss of interest, often accompanied by neurovegetative and neurocognitive symptoms, like insomnia, fatigue, weight loss, and impaired cognition (Reed et al., 2019). In severe cases, depressive disorder may even be able to increase the risk of somatic diseases like stroke and cancer, as well as suicide (Otte et al., 2016). World Health Organization (WHO) has ranked depressive disorder as the third leading cause of the years lived with disability (YLD) in the Global burden of Disease (GBD) study 2017 (GBD, 2018). In China, the recent nationwide epidemiological survey of mental disorders also reported mood disorders had become the second most prevalent mental disease with a lifetime prevalence of 7.4%, in which depressive disorder accounted for 6.8% (Huang et al., 2019). However, the specific biomarkers for clinical diagnosis of depressive disorder were still in lack and its biological mechanisms were not well clarified. Traditional mechanisms like monoamine‐depletion hypothesis can not fully explain the pathogenesis of depressive disorder (Malhi & Mann, 2018). Therefore, more promising research are needed to explore new underlying mechanisms.

Research over the recent decades have indicated that inflammation might be involved in the onset and development of depressive disorder (Beurel et al., 2020). Overactivation of inflammatory responses and increased levels of inflammatory mediators like interleukin (IL)−1β, IL‐6, tumor necrosis factor alpha (TNF‐α), the acute phase reactant (CRP), and high mobility group box 1 (HMGB1) were seen in both patients and rodents with depressive disorder (Cernackova et al., 2020; Liu et al., 2019; Xie et al., 2021; Zolfaghari et al., 2021). Our previous work focused mainly on the role of HMGB1, a late mediator of inflammation in depressive‐like behaviors (Wang et al., 1999). We first discovered that HMGB1 was significantly increased in mice under acute or chronic stress, while application of glycyrrhizinic acid (GZA), an inhibitor of HMGB1, could markedly improve depressive symptoms (Lian et al., 2017; Wu et al., 2015). These findings were later demonstrated by other teams (Fu et al., 2019; Yang et al., 2020). The increased HMGB1 might subsequently induce the imbalance of two different metabolic approaches in kynurenine pathway (KP) in microglia and astrocytes, thus resulting in the abnormal glutamatergic neurotransmission and contributing to the development of depressive‐like behaviors (Wang, Lian, Dong et al., 2018; Wang, Lian, Su et al., 2018; Wang, Lian et al., 2019). It was interesting that our results also showed the increased HMGB1 mainly originated from neurons and microglia in the central nervous system (CNS) (Wang, Huang et al., 2020), which then brought out important questions about how HMGB1 communicated among cells and the possible mechanisms.

There was evidence that the release of HMGB1 could be regulated by connexins (Cxs). Cxs are fundamental structures of intracellular communications, which can form two functional forms, namely gap junctions (GJ) and hemichannels (HC) (Takeuchi & Suzumura, 2014). In a mouse model of acute lung injury (ALI), application of a broad‐spectrum Cxs blocker named P5 significantly reduced the endotoxin‐induced release and accumulation of HMGB1 (Wang et al., 2020). Recent evidence also strongly indicated that Cxs dysfunction might be involved in the pathogenesis of depressive disorder (Ren et al., 2018). In a study of depressive‐like behaviors in mice induced by inescapable stress, knock‐down of Cxs could result in significant anti‐depressant effects as well as the improvement of cognitive functions (Quesseveur et al., 2015). Similarly, injection of a nonselective Cxs inhibitor named carbenoxolone (CBX) into the hippocampus of mice exhibited the same therapeutic effects in a depression model of CMS (Chen, Wang, Zuo et al., 2016). Therefore, it is probable that abnormal expressions or functions of Cxs are involved in the development of depressive disorder. On these foundations, we conjectured that Cxs might be involved in HMGB1 signaling during the process of depressive‐like behaviors. Since there is coexpression of Cx36 in both neurons and microglia, (Medina‐Ceja et al., 2019; Orellana et al., 2009) and based on our previous work, we mainly focus on the role of Cx36 in HMGB1 mediated depressive‐like behaviors.

Actually, Cx36 is the main connexin between neurons, which not only plays an important role in the development of neurons, but also participates in glutamate‐induced neuronal excitotoxicity and injury (Arundine & Tymianski, 2004; Belousov et al., 2017). Since neuronal injury, glutamate excitotoxicity, and enhanced HCs activity were closely related to the development of depressive disorder, we further speculated that Cx36 might be the mediator in the process of HMGB1 mediated depressive‐like behaviors based on our previous work. However, the underlying mechanisms were not fully elucidated. In this study, we sought to explore the change of Cx36 under chronic unpredictable mild stress (CUMS) and its role in HMGB1 signaling.

## 2 METHODS

### 2.1 Animals and treatments

All the animals used in this study were 8‐week‐old male BALB/c mice purchased from the experimental animals center (Second Military Medical University, Shanghai, China). The mice were raised in standard conditions of constant temperature (21–23°C) and humidity (50%−52%) on a light/dark cycle of 12 h with food and water available ad libitum. All experiments were conducted in accordance with the standards established by the experimental animal ethics committee of Second military Medical University. Before the experiments, mice could adapt to new feeding conditions for 2 weeks. In the experiment of GZA (G2137, Sigma‐Aldrich, USA) treatment, mice were randomly assigned to four groups, namely control group, CUMS group, CUMS+GZA group, and GZA group. In the experiment of quinine (V900715, Sigma‐Aldrich, USA) treatment, mice were randomly assigned to four groups, namely control group, CUMS group, CUMS+quinine group, and quinine group. GZA and quinine were dissolved in 0.9% sterile saline and injected intraperitoneally every day from the day before CUMS. According to previous studies (Nassiri‐Asl et al., 2009; Wang, Lian, Dong et al., 2018), GZA was applied at a dose of 20 mg/kg and quinine at 50 mg/kg. The final volume of all drugs injected into each mouse was 200 μl. Mice of control and CUMS group were intraperitoneally administered of equal volume of saline every day.

### 2.2 Chronic unpredictable mild stress

According to our previous studies (Leng et al., 2018; Lian et al., 2017), the following mild stimuli were applied randomly to mice: (i) 5 min of exposure to high temperature (45°C); (ii) 10 min of cage shaking; (iii) 1 h of physical restraint; (iv) overnight illumination; (v) 14 h of cage tilt (45°); (vi) 12 h of water deprivation; (vii) 12 h of food deprivation; (viii) 1 h of exposure to noise (86 dB). These stressors were alternately scheduled for 4 weeks.

### 2.3 Behavioral tests

After four‐week stimuli, behavioral tests were performed in mice to evaluate depressive‐like behaviors. All the tests were conducted between 18:00 and 21:00.

#### 2.3.1 Sucrose preference test

By reference to previous studies (Lian et al., 2017; Wang et al., 2018), sucrose preference test (SPT) was conducted to assess the affection of mice for sweets, which reflected anhedonia, one of the key symptoms in depressed patients. Mice first learned to choose 1% sucrose solution or water from two bottles for 2 weeks. During this period, bottles were switched every 3 days to avoid preference for either side. Sucrose preference was measured before and after 4 weeks of CUMS. Mice were deprived of food and water for 12 h, and then allowed free access to 1% sucrose and water in two separate bottles. An hour later, the bottles were weighed and sucrose preference was calculated. Sucrose preferences (%) = sucrose consumption/(sucrose+water consumption) × 100%.

#### 2.3.2 Tail suspension test

Tail suspension test (TST) was conducted to asess despair and helplessness in depressed mice (Cryan, Mombereau, Vassout, 2005). The immobility time of mice were measured by Tail Suspension PHM‐300 (Med Associates Inc., USA). Briefly, a piece of medical tape was wrapped around the mouse tail at about 1 cm from the tail tip. Then the tape was fixed on the hook of the instrument to let mice hang upside down in the dark box. The test lasted for 6 min and the immobility time of final 5 min was recorded (lower threshold value of 0.5).

#### 2.3.3 Forced swimming test

Forced swimming test (FST) was conducted to assess despair in depressive‐like behaviors (Cryan & Holmes, 2005; Cryan & Slattery, 2007). Briefly, mice were gently put into a transparent cylindrical container (10 cm in diameter and 25 cm in height) with warm water (25 ± 1°C). The water depth was about 16–18 cm. Mice's activities were recorded by video tracking equipment (Shanghai Xinruan Info Technology Co., Ltd, China). The whole process lasted 6 min and the immobility time of the final 5 min was recorded.

#### 2.3.4 Open field test

Open field test (OFT) was conducted to assess the spontaneous activities of mice (Yang et al., 2015), which often reflected the anxious performance of mice (Chen, Wang, Liang et al., 2016). During the test, mice were put gently into the dark box (RD1112‐IOF, Shanghai Transfer Info Technology Co., Ltd, China) with a size of 710 mm (width) × 710 mm (length) × 1460 mm (height). The activites of mice were video tracked by an infrared detector. After 6 min of test, the central and total distance of each mice were calculated by OpenField2.8.7 software (Shanghai Transfer Infor Technology Co., Ltd, China).

### 2.4 Tissue preparation

After behavioral tests, mice were all sacrificed and tissues were quickly obtained.

#### 2.4.1 For western blot analysis

The hippocampus and prefrontal cotex were weighed and homogenized in the lysis buffer (P0033, Shanghai Beyotime Biotechnology Co., Ltd, China) at a concentration of 160 μl/mg. The homogenate was placed on ice for 40 min and then centrifuged at 12,000 rpm for 20 min at 4°C. The supernatant was transferred into a new centrifuge tube for later use and the excess samples were stored at −80°C.

#### 2.4.2 For immunofluorescence

The hippocampus was fixed in 4% paraformaldehyde for 24–48 h, and dehydrated in 30% sucrose solution for at least 24 h until the samples sunk to the bottom. The samples were stored at 4°C for later use.

#### 2.4.3 For enzyme‐linked immunosorbent assay

The hippocampus was weighed and homogenized in PBS (pH 7.2–7.4) with protease inhibitors (1 μg/L, P1005, Shanghai Beyotime Biotechnology Co., Ltd, China) at a concentration of 160 μl/mg. Then the homogenate was centrifuged at 12,000 rpm for 20 min at 4°C and the supernatant was obtained for later use. The excess samples were stored at −80°C.

The blood samples were collected by eyeball extirpating under light anaesthesia. The samples were placed at room temperature for 20 min and then centrifuged at 12,000 rpm for 20 min at 4°C. The serum was obtained and transferred into new centrifuge tubes for later use. The excess samples were stored at −80°C.

### 2.5 Western blot analysis

The protein concentration of hippocampus and prefrontal cortex was determined by a BCA kit (P00125, Shanghai Beyotime Biotechnology Co., Ltd, China). Samples containing 30 μg of protein were mixed with loading buffer and denatured in boiling water for 10 min. The proteins were separated by 12.5% SDS‐PAGE gels and then eletrically transferred onto polyvinylidene difluoride membranes (PVDF, Millipore, USA). After that, membranes were immersed into TBST solution (TBS containing 0.1% Tween‐20) with 5% bovine serum albumin (BSA) and blocked for 1 h at room temperature. Then membranes were incubated in different primary antibodies (anti‐tubulin: 1/1000, #5568, CST, USA; anti‐Cx36: 1/1000, sc‐398063, Santa Cruz, USA) overnight at 4°C. On the next day, membranes were washed with TBST solution for three times and incubated in appropriate secondary antibodies (goat anti‐rabbit IgG: 1/1000, A0208; goat anti‐mouse: 1/1000, A0216; all from Shanghai Beyotime Biotechnology Co., Ltd, China) for 1 h at room temperature. Finally, bands were detected by Biorad imaging system, and the intensities were analyzed by Image J software.

### 2.6 Immunofluorescence (IF)

The samples of hippocampus were dried and embedded with optimum cutting temperature (OCT) compounds and frozen at −20°C. The samples were cut into slices of 10 μm using a Leica CM‐1900 slicer. Then sodium citrate solution (PH 6.0) was added for antigen repairing and slices were boiled in the water bath at 95°C for 10 min. After washed with phosphate buffered solution (PBS), slices were incubated with primary antibodies (anti‐Cx36: 1/200, sc‐398063; anti‐Iba1: 1/200, #17198, CST, USA; anti‐GFAP: 1/200, #80788, CST, USA; anti‐NeuN: 1/500, #12943, CST, USA) overnight at 4°C. On the next day, slices were incubated with secondary antibodies (anti‐rabbit IgG: 1/200, #711‐095‐152; anti‐mouse IgG: 1/400, #715‐165‐150; all from Jackson ImmunoResearch, USA) for 1 h at room temperature. After that, slices were incubated with DAPI (1/1000, CST, USA) at room temperature for 5 min. Fluorescent images were taken by fluorescent microscope (Carl Zeiss, Germany) and neurons were counted using Nikon Imaging Elements Software (USA).

### 2.7 Enzyme‐linked immunosorbent assay (ELISA)

The levels of HMGB1, TNF‐α, and IL‐1β in the serum and hippocampus were measured by ELISA kits bought from Shanghai Westang Biotech (China) according to the manufacturer's instructions. Briefly, the serum and hippocampal protein extracts were diluted five or 10 times with the sample buffer. Then add the standards and samples into the 96‐well coated plate with 100 μl per well. The levels of HMGB1, TNF‐α, and IL‐1β were measured using the double antibody sandwich method. After the immunoreactions, the absorbance value at 450 nm was measured within 30 min and the concentration of cytokines was calculated according to the optical density (OD) values. The sensitivity of these kits was 2 ng/ml for HMGB1, 4 pg/ml for TNF‐α, and 8 pg/ml for IL‐1β.

### 2.8 Primary neuronal cultures

The isolation and culture of hippocampal neurons from postnatal (P0‐P1) mice were referred to previous studies (Beaudoin et al., 2012; Gardner et al., 2012; Kaech & Banker, 2006). Briefly, mice were first disinfected with 75% alcohol thoroughly and euthanized by decapitation. The brains were quickly isolated and placed in a 60 mm dish containing dissection medium. The hippocampus was gently separated under a dissecting microscope, and the meninges were removed as completely as possible. The hippocampus was transferred into a new dish with 1 ml of fresh dissection medium and cut into pieces with fine scissors. Then two drops of DNase solution (DN25, Sigma‐Aldrich, USA) and 1 ml of 0.25% trypsin solution (#25300‐054, Gibco, USA) were added into the dish. The tissues were incubated at 37°C in a cell culture incubator for 10 min and gently shaked every 5 min. After that, 2 ml of plating medium (Neurobasal‐A containing 2% B27; #21103‐049 and #12587‐010, Gibco, USA) was immediately added to inactivate DNase and trypsin. After centrifuged at 800 rpm for 5 min, the sediment was resuspended in 3 ml of plating medium and gently dissociated to obtain a homogenous cell suspension. The viable cell density was estimated on a hemocytometer and the suspension was plated on 60‐mm diameter dishes coated with poly‐L‐lysine (P9155, Sigma‐Aldrich, USA). Then the neurons were incubated in the cell culture incubator at 37°C for 4–6 h.

### 2.9 Whole‐cell patch‐clamp recording

The electrophysiological signalings were recorded by Clamp 700B amplifier (Axon, USA) and transformed by Digidatal 1440 (Axon, USA) at room temperature. The procedures were referred to previous reports (Due et al., 2012; Gomez et al., 2019). Briefly, the plating medium in the dish was switched into artificial cerebrospinal fluid (ACSF, 32°C) containing (in mM): 125 NaCl, 25 NaHCO_3_, 1.25 NaH_2_PO_4_, 2.5 KCl, 2 CaCl_2_, 1.5 MgCl_2_, 10 D‐glucose, adjusted to pH 7.4, and osmolarity 300 mosM. The dish was continuously perfused with ACSF at a rate of 1–2 ml/min. Electrodes (3–5 MΩ) were filled with recording solution containing (in mM): 112 KCl, 2 MgCl_2_, 0.1 CaCl_2_, 11 EGTA, 10 HEPES, with pH adjusted to 7.2 and osmolarity to 300 mosM and controlled by a micromanipulator. When tight sealing between neurons and electrodes was forged, breakage of neuronal membranes was quickly conducted, and properties of neurons were written down like resting membrane potential (RMP). Under whole‐cell patch current clamp, single action potential (AP) and AP series were recorded. The firing frequency of APs was recorded in response to injecting current steps (50–300 pA/500 ms) and the kinetic properties of APs were analyzed including amplitude, half‐width, threshold, and rheobase. Under whole‐cell patch voltage clamp, elicited currents were recorded in response to voltage steps (−90 to −50 mV). During the recording, drugs were applied by a pressure‐driven eight‐channel delivery system (ALA Scientific, USA). LPS (1 μg/ml), GZA (50 μM), and quinine (200 μM) were all dissolved in ACSF, and administered alone or in combination according to previous studies (Due et al., 2012; Gomez et al., 2019). Data acquired was analyzed by pCLAMP10.0 software (Axon, USA).

### 2.10 Statistical analysis

All data were expressed as mean ± standard error of the mean (SEM). One‐way or two‐way analysis of variance was performed for comparison among multiple groups followed by a least significant difference (LSD‐t) post hoc test. Nonparametric test (Kruskal–Wallis test, K–W test) was adopted under nonnormal distribution or heterogeneity of variance among groups. The difference was considered statistically significant when *p* < .05. All statistical analyses were performed with SPSS 22 (SPSS, USA).

## 3 RESULTS

### 3.1 Changes of Cx36 in HMGB1 mediated depressive‐like behaviors induced by CUMS

#### 3.1.1 Cx36 was increased by CUMS, while decreased by GZA

**FIGURE 1:**
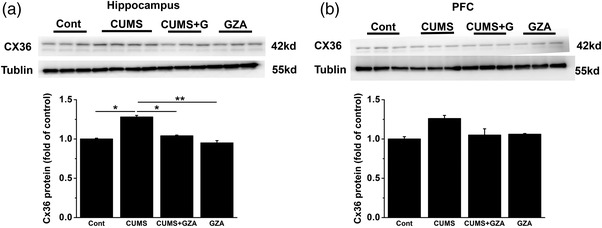
GZA decreased the expression of Cx36 in hippocampus induced by CUMS. (a) Expression of Cx36 in hippocampus was significantly increased by CUMS, while decreased after treatment with GZA (*p < .05, **p < .01, *n* = 6–8 for each group). (b) There was no significant difference of Cx36 expression among groups (*p* > .05, *n* = 6–8 for each group). GZA, glycyrrhizinic acid; Cx36: Connexin36; CUMS: chronic unpredictable mild stress

Evidence indicated that Cx36 took part in several pathophysiological activities, like injury and inflammation (Wang et al., 2019), which was closely related to depressive disorder. Therefore, we first sought to see how Cx36 changed in depressive model induced by CUMS. After 4‐week chronic stress, behavioral tests including SPT, TST, FST were performed to confirm whether depressive model was successfully constructed. As shown in Figure S1a–c, mice in CUMS group displayed significantly decreased sucrose preference and prolonged immobility time, which were important features of depressive disorder. After treatment with GZA, the above depressive symptoms were obviously relieved (Figure S1a, *F* = 5.606, *p* = .003, analysis of variance; Figure S1b, *F =* 2.740, *p* = .059, analysis of variance; Figure S1c, *H =* 17.574, *p* = .001, K–W test). GZA was a Chinese herbal extract known as the direct inhibitor of HMGB1. Our results showed that GZA could not only inhibit HMGB1 in both serum and hippocampus, but also reduce downstream cytokines like TNF‐α and IL‐1β (Figure S2a, *F* = 5.182, *p* = .006, analysis of variance; Figure S2b, *F* = 6.568, *p* = .002, analysis of variance; Figure S2c, *H* = 18.218, *p* < .001, K–W test; Figure S2d, *F* = 9.738, *p* < .001, analysis of variance; Figure S2e, *H* = 17.294, *p* = .001, K–W test; Figure S2f, *H* = 18.227, *p* < .001, K–W test). After that, Cx36 proteins in the hippocampus and prefrontal cortex (PFC) were measured. Interestingly, the expression of Cx36 was signicantly increased in CUMS group in the hippocampus but not PFC, while application of GZA almost totally reversed the increase, (Figure 1a, *F* = 5.405, p = .021; Figure 1b, *F* = 0.582, *p *= .643, analysis of variance) which suggested that overexpression of Cx36 in the hippocampus might be involved in the nflammation of depressive‐like behaviors induced by chronic stress.

#### 3.1.2 Cx36 in hippocampal neurons was increased by CUMS, while decreased by GZA

**FIGURE 2:**
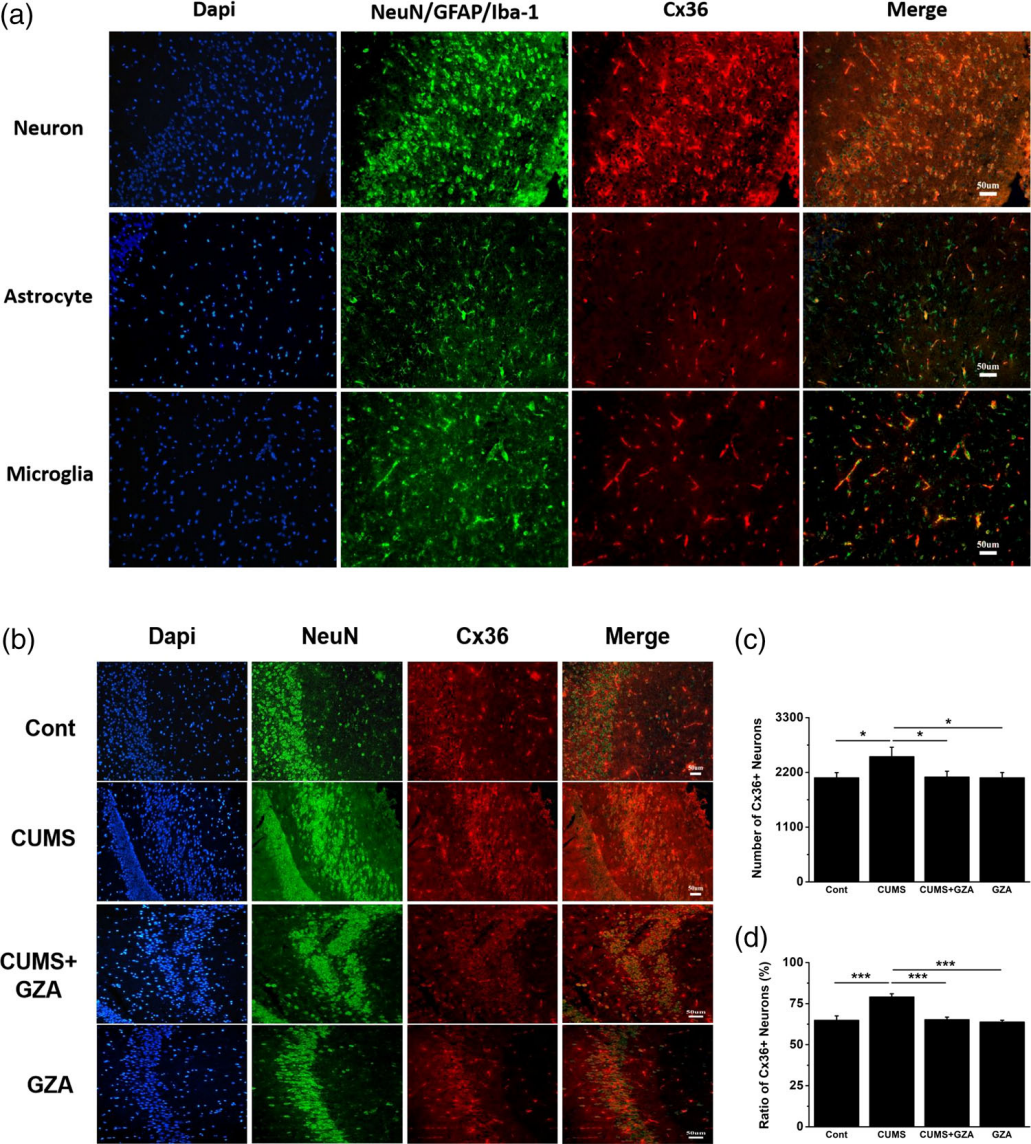
Changes of Cx36 in hippocampal neurons of mice exposed to CUMS and GZA. (a) Cx36 (red) was colabeled with neurons (NeuN, green), astrocytes (GFAP, green), microglia (Iba‐1, green), and nuclei (DAPI, blue) in hippocampal slices of control mice. Cx36 was expressed in hippocampal neurons and microglia, but not in astrocytes (Scale bar: 50 μm). (b) Cx36 (red) was colabeled with neurons (NeuN, green) and nuclei (DAPI, blue) in the hippocampus of different groups (Scale bar: 50 μm). The quantity (c) and proportion (d) of Cx36 positive neurons in the hippocampus were both significantly increased by CUMS while decreased after treatment with GZA (*p < .05, ***p < .001, *n* = 6 for each group). GZA Glycyrrhizinic acid; Cx36, Connexin36; CUMS, chronic unpredictable mild stress; NeuN, neuronal nuclei antigen; GFAP, Glial Fibrillary Acidic Protein; Iba‐1, ionized calcium binding adapter molecule 1; DAPI, 4,6‐diamidino‐ 2‐phenylindole

Since the expression of Cx36 in hippocampus was greatly changed, we sought to further nflamm its cellular localization using triple‐labeling immunofluorescence. Neurons, astrocytes and microglias were labelled in green by NeuN, GFAP, and Iba‐1, respectively. Nuclei were labelled in blue by dapi, and Cx36 in red by its specific antibody. As shown in Figure 2a, Cx36 was mainly expressed in neurons and microglia, while hardly expressed in astrocytes. This was consistent with other studies (Dobrenis et al., 2005; Medina‐Ceja et al., 2019). In order to see how Cx36 changed in hippocampal neurons after different treatments, immunofluorescence continued to be adopted as mentioned above. Neurons were labeled in green, Cx36 in red, and nuclei in blue (Figure 2b). The results based on cell‐counting showed the absolute quantity and proportion of Cx36 positive neurons were both increased significantly in CUMS group, while decreased markedly after GZA treatment. Treatment with GZA alone made no difference (Figure 2c, *F* = 2.403, *p* = .098, analysis of variance; Figure 2d, *H* = 12.833, *p* = .005, K–W test).

### 3.2 Inhibition of Cx36 by quinine could alleviate depressive‐like behaviors induced by CUMS

#### 3.2.1 Qunine alleviated depressive‐like behaviors induced by CUMS

**FIGURE 3:**
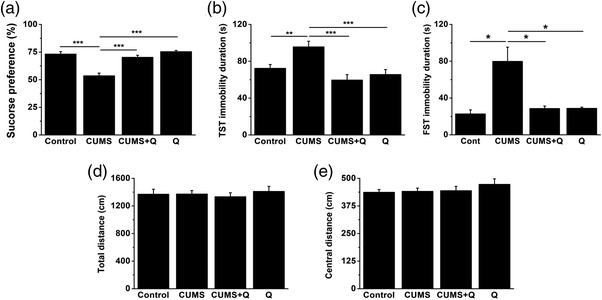
Quinine alleviated depressive‐like behaviors induced by CUMS. CUMS significantly decreased the sucrose preference (a) and extended the immobility duration of mice in TST (b) and FST (c), while treatment with quinine could markedly alleviate these symptoms (a–c) (*p < .05, **p < .01, ***p < .001, *n* = 8–10 for each group). However, there was no significant difference in the total distance (d) and central distance (e) among the treated groups in OFT (*p* > .05, *n* = 8–10 for each group). GZA, Glycyrrhizinic acid; Cx36, Connexin36; CUMS, chronic unpredictable mild stress; TST, tail suspension test; FST, forced swimming test; OFT, open field test

On the basis of the above findings, the depressive‐like behaviors of mice induced by chronic stress could be obviously alleviated by inhibition of HMGB1 by GZA, which might be through down‐regulation of Cx36. Therefore, we went on to see whether direct inhibition of Cx36 by its specific inhibitor, quinine, could play such a therapeutic role. After 4‐week stress, behavioral tests were performed including SPT, TST, FST, and OFT. According to the results, CUMS led to significantly lower sucrose preference and extended immobility time during SPT, TST, and FST, while application of quinine could markedly alleviate those symptoms. Treatment with quinine alone made no difference (Figure 3a, *H *= 24.250, *p* < .001, K–W test; Figure 3b, *F* = 7.823, *p* < .001, analysis of variance; Figure 3c, *H *= 14.609, *p* = .002, K–W test). OFT was adopted to observe the spontaneous activities of mice in the novel environment, and often used to determine whether there was anxiety‐like behaviors (Dobrenis et al., 2005; Medina‐Ceja et al., 2019). In our study, there were no significant differences in the central or total distance among groups, which indicated anxiety‐like behaviors were absent.(Figure 3d, *H *= 0.457, *p* = .928; Figure 3e, *H =* 0.291, *p* = .962, K–W test)

#### 3.2.2 Qunine decreased pro‐inflammatory cytokines in the serum and hippocampus induced by CUMS

**FIGURE 4:**
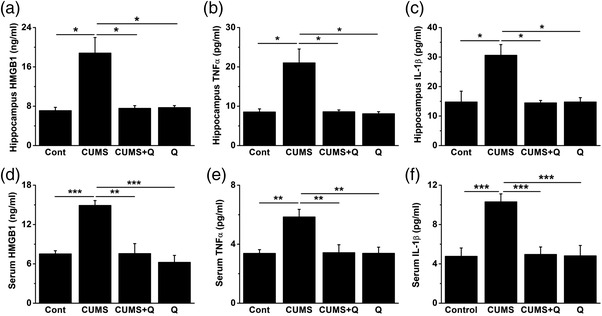
Quinine decreased the release of pro‐inflammatory cytokines in serum and hippocampus induced by CUMS. The hippocampal levels of HMGB1 (a), TNF‐α (b), and IL‐1β (c) were significantly increased by CUMS, but decreased significantly after treatment with quinine (a–c) (*p < .05, *n* = 8–10 for each group). Similarly, the serum levels of HMGB1 (d), TNF‐α (e), and IL‐1β (f) were also increased significantly by CUMS and decreased after quinine administration (*p < .05, **p < .0l, ***p < .001, *n* = 8–10 for each group). CUMS, chronic unpredictable mild stress; HMGB1, high mobility group box 1; TNF‐α, tumor necrosis factor alpha; IL‐1β, interleukin‐1β

According to our findings, inhibition of Cx36 by quinine could play a therapeutic role in depressive‐like behaviors induced by CUMS. In addition, studies have indicated that cytokines could be regulated by Cx36 (Wang et al., 2020). Therefore, to further investigate the possible underlying mechanisms, ELISA was adopted to detect three classical proinflammatory cytokines in the serum and hippocampus of mice, which might be associated with our study, namely HMGB1, TNF‐α, and IL‐1β. As shown in Figure 4, either in the serum or hippocampus, the levels of HMGB1, TNF‐α and IL‐1β were all significantly increased in CUMS group, while treatment of quinine markedly decreased them. Application of quinine alone made no difference (Figure 4a, *H* = 18.914, *p* < .001, K–W test; Figure 4b, *H* = 10.566, *p* = .014, K–W test; Figure 4c, *H* = 17.027, *p* = .001, K–W test; Figure 4d, *H* = 17.217, *p* = .001, K–W test; Figure 4e, *H* = 13.099, *p* = .004, K–W test; Figure 4f, *F* = 10.104, *p *< .001, analysis of variance). These results suggested that inhibition of Cx36 was likely to prevent the proinflammatory cytokines from releasing, thus alleviating depressive‐likex behaviors.

### 3.3 Inhibition of HMGB1 and Cx36 altered the electrical activities of hippocampal neurons induced by LPS

Since depressive‐like behaviors were ultimately related to the electrical activities of neurons and their survival, we sought to explore whether GZA and quinine could improve such activities. In order to obtain neurons approximating the in vivo ones as closely as possible, we chose primary hippocampal cultures. Whole‐cell patch clamping was adopted to record the electrical activities of hippocampal neurons in different groups. Here we used lipopolysaccharide (LPS) to simulate the inflammatory environment of neurons in vivo. Experimental evidence has shown that LPS was able to induce acute depressive‐like behaviors, and could elevate several pro‐inflammatory cytokines including HMGB1, TNF‐α, IL‐1β, and so on (Aguilar‐Valles et al., 2015; Walker et al., 2019). Therefore, we chose LPS to investigate the changes of neuronal excitability and currents induced by stress, which were represented by action potentials (Aps) and inward currents.

**FIGURE 5:**
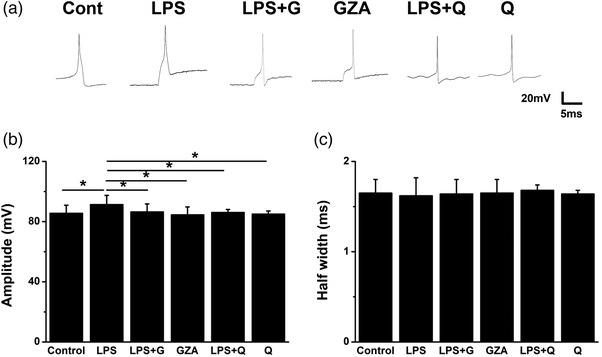
Quinine and GZA reduced the amplitude of single action potential induced by LPS in hippocampal neurons. (a) The Action potential waveforms of hippocampal neurons from different groups. (b) The amplitude of action potentials was significantly increased by LPS, while decreased after treatment with GZA or quinine (*p < .05, *n* = 8 for each group). (c) There was no significant difference in half width of action potentials among different groups (*p* > .05, *n* = 8 for each group). GZA, Glycyrrhizinic acid; LPS, lipopolysaccharide

First, the single action potential was recorded (Figure 5a). The amplitude and half width of each neuron was measured and analyzed. Compared with the control group, LPS could significantly increase the amplitude of the action potential, while application of GZA or quinine could both restore it. Treatment with GZA or quinine alone made no difference (Figure 5b, *F* = 2.873, *p* = .012, analysis of variance). As to the half width of the action potential, there was no significant change among all the groups (Figure 5c, *H *= 0.424, *p *= .995, K–W test).

**FIGURE 6:**
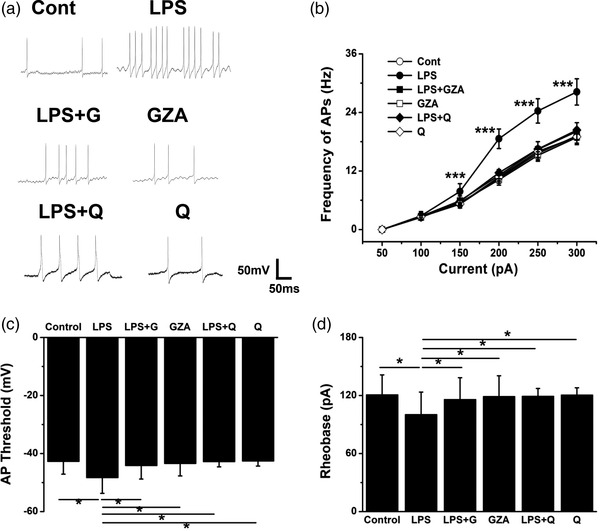
Qunine and GZA reduced the excitability of hippocampal neurons induced by LPS. (a) The representative voltage response traces of hippocampal neurons from different groups. (b) The firing frequency of hippocample neurons was significantly increased by LPS with the injecting current of 150–300 pA, while decreased after treatment with GZA or quinine (***p < .001, *n* = 8 for each group). In addition, the AP threshold (c) and rheobase (d) of hippocampal neurons were both decreased by LPS, but recovered after treatment with GZA or quinine (*p < .05, *n* = 8 for each group). GZA, Glycyrrhizinic acid; LPS, lipopolysaccharide

Then, the firing frequency of action potentials was recorded (Figure 6a). When the frequency was plotted against injected current steps, there were significant differences among groups. LPS could markedly increase the firing frequency within the current range of 150—300 pA, while treatment with GZA or qunine could significantly decrease it. Application of GZA or quinine alone made no difference (Figure 6b, *F* = 103.861, *p* < .001, analysis of variance). Similarly, the threshold and rheonobase current of the action potentials were all decreased when mice were exposed to LPS treatment, while application of GZA or quinine could significantly increase them. There was no significant change when GZA or quinine was applied alone (Figure 6c, *F* = 2.806, *p* = .013; Figure 6d, *F* = 2.501; *p* = .021, analysis of variance).

**FIGURE 7:**
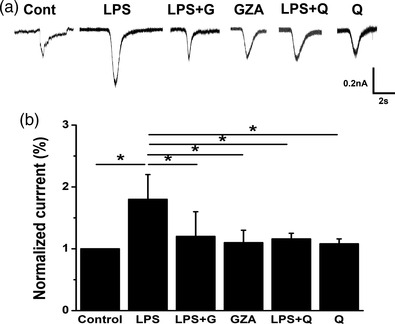
Qunine and GZA attenuated the inward current of hippocampal neurons induced by LPS. (a) The representative current traces of hippocampal neurons from different groups. (b) The inward current was significantly increased by LPS, while decreased after treatment with GZA or quinine (*p < .05, *n* = 8 for each group). GZA, glycyrrhizinic acid; LPS, lipopolysaccharide

Since the change of Aps was often due to the flux of ions and intracellular ion overload would lead to neuronal excitatory damages, the inward currents of hippocampal neurons were recorded afterwards (Figure 7a). Exposure to LPS could significantly increase the inward current of hippocampal neurons, while application of GZA or quinine obviously decreased the current. There was no significant change when GZA or quinine was applied alone (Figure 7b, *H* = 17.213, *p* = .004, K–W test). These results suggested that the excitability of hippocampal neurons was remarkably increased under stress, which might take part in the pathophysiology of depressive‐like behaviors. Besides, inhibition of HMGB1 or Cx36 could play a neuroprotective role through mitigation of such excitability.

## 4 DISCUSSION

Our findings indicated that the increase of Cx36 might be involved in HMGB1‐mediated depressive‐like behavious induced by CUMS. Inhibition of HMGB1 might alleviate depressive symptoms through down‐regulation of Cx36 and subsequent inflammatory cytokines like TNF‐α and IL‐1β, as well as reducing the neuronal excitotoxicity. Furthermore, inhibition of Cx36 could also mitigate depressive symptoms which might be through blocking the release of central and peripheral proinflammatory cytokines like HMGB1, TNF‐α, IL‐1β, and reducing the neuronal excitotoxicity as well. Interestingly, there might be a strong mutual interaction between HMGB1 and Cx36 in the development of depressive‐like behaviors induced by CUMS.

So far, many hypotheses have been proposed about the etiology and pathophysiology of depressive disorder, however, no single one could perfectly explain all aspects of the disease. Dysfunction of Cxs was a relatively new hypothesis and might act as bridges among those mechanisms (Ren et al., 2018). Evidence from animal studies of depressive disorder and autopsies of patients with major depressive disorder or suicidal behaviors showed overexpression or dysfunction of Cxs in neuroglial cells in the CNS and antidepressants could cure these abnormalities (Belousov et al., 2017). Cxs might block the propagations of spreading depressions by promoting the uptake of glutamate and K^+^, thus leading to neuronal inactivation. These results were consistent with our findings. It was the first time to discover that expression of Cx36 was increased significantly in the hippocampus of mice exposed to CUMS, while treatment with GZA obviously improved depressive symptoms through down‐regulation of Cx36, suggesting that Cx36 might play a destructive role in depression. Interestingly, GZA was an inactive analogue of CBX, the nonselective inhibitor of gap junctions. The drug itself could not bind to Cxs to exert biological effects (Mitrou et al., 2016; Ozog et al., 2002a; Patrone et al., 2014). However, according to our previous work, GZA could alleviate depressive‐like behaviors of mice through inhibition of HMGB1 (Wang et al., 2018). Evidence also indicated that GZA might directly bind to HMGB1 or form DNA complex so that the nuclear translocation or release of HMGB1 was obstructed (Bailly & Vergoten, 2020). In this way, HMGB1 could not interact with its receptors like advanced glycation end products receptors (RAGE), toll‐like receptors (TLR) 2 and TLR4, which also prevented the follow‐up increase of other inflammatory cytokines like TNF‐α and IL‐1β (Lian et al., 2017). Hence, the physiological activity of HMGB1 was decreased and GZA exerted anti‐inflammatory effects (Bailly & Vergoten, 2020). Therefore, we conjectured that increase of Cx36 in CUMS might be mediated by HMGB1. This was also seen in other studies that expression of Cx36 was significantly elevated by injection of capsaicin, CFA or IL‐6 in different models of injury, while inhibition of Cx36 could alleviate the inflammatory symptoms and motor deficits (Garrett & Durham, 2008; Wang et al., 2019). However, in a study of cytokine‐induced oxidative stress, pro‐inflammatory cytokines like IL‐1β, TNF‐α, and IFN‐γ could markedly down‐regulate the expression of Cx36 through activation of AMPK (Allagnat et al., 2013). These obviously contradictory results might be probably due to the different functional constructions of Cx36, namely GJs and HCs (Quesseveur et al., 2015). Studies indicated that the roles of HCs and GJs in depressive disorder might be totally opposite. Promotion of GJs communication between neuroglial cells might increase neurotrophic supplies and enhance brain plasticity, thus playing an antidepressant role (Jeanson et al., 2016). However, enhanced activities of HCs might result in the increase of glutamate‐induced excitotoxicity and neural injuries (David et al., 2009; Hanstein et al., 2009; Orellana et al., 2015; Rainer et al., 2012). Although Cx36 inhibitors or gene‐editing of Cx36 were found to alleviate or exacerbate sick behaviors in different animal models, their effects on Cx36 protein itself or the functions of HCs and GJs were not yet clear. In our study, the functions of Cx36 HCs and GJs were not covered as well, so we were going to arrange more researches to further explore the underlying mechanisms.

Both clinical and experimental evidence showed that the emotional circuits associated with depressive disorder consisted of different parts of the brian, such as PFC, ventral hippocampus, amygdala and nucleus accumbens (Nac) (Wang, Wang et al., 2021). In our study, we found the increase of Cx36 induced by chronic stress mainly appeared in the hippocampus, but not PFC. One possibility was that the expression of Cx36 might be time‐ and region‐dependent. In a study of an inflammatory CNS disease, the expression of Cx36 began to change in the hippocampus four weeks after inflammation was induced, while it did not change in the cortex until 8 weeks later (McCracken et al., 2005). Therefore, the findings that Cx36 in PFC did not change obviously under chronic stress might be due to the limited duration of the experiment. Although Cx36 was expressed in both the hippocampus and PFC (McCracken et al., 2005), studies on the role of Cx36 in neuropsychiatric diseases focused mainly on hippocampus rather than PFC. More evidence was needed to discuss the changes of Cx36 in PFC.

Evidence showed that Cx36 was expressed mainly in neurons and microglia, while hardly in astrocytes or other immunocytes, (Dobrenis et al., 2005; Nagy & Rash, 2017) which was consistent with our immunohistochemical results. Furthermore, Cx36 might be expressed only in a minority of neurons such as interneurons in hippocampus and thalamus, sparse neurons in new cortex. In these neurons, Cx36 formed electrical synapses for messaging, while in most other neurons, chemical synapses were predominate (Mesnil et al., 2021). It was also found that Cx36‐positive neurons were mostly GABA‐ergic inhibitory nflammation, which might be an important cellular basis in Cx36‐mediated depressive‐like behaviors (Hamzei‐Sichani et al., 2012; Köster‐Patzlaff et al., 2009). Furthermore, GABA‐ergic neurons were demonstrated to be highly activated in CUS‐induced depressive‐like behaviors, while inhibition of their activities could play an antidepressant role (Wang et al., 2021). Therefore, Cx36 might promote depressive‐like behaviors through enhancing the excitability of GABA‐ergic neurons. However, the exact role of GABA‐ergic neurons in depressive disorder was disputed and needed more researches to confirm.

Since the increase of Cx36 might aggravate depressive‐like behaviors and GZA could not block Cx36 directly, we then sought to choose a relatively specific inhibitor, namely quinine. Quinine was reported to be the inhibitor of both Cx36 and Cx50. However, Cx50 was hardly expressed in mammals and only seen in lens (Srinivas et al., 2001). Hence, quinine was applied as the specific inhibitor of Cx36 (Faridkia, Yaghmaei, Nassiri‐Asl, 2016; Nassiri‐Asl, Zamansoltani, Torabinejad, 2009). In studies of epilepsy and migraine, quinine was found to be able to alleviate cognitive impairments and headache through down‐regulation of Cx36, which was consistent with our findings that quinine could alleviate depressive‐like behaviors induced by CUMS (Gajda et al., 2005; Nassiri‐Asl et al., 2008; Sarrouilhe et al., 2014). However, there were also findings that inhibition of Cx36 by quinine or knockout of Cx36 could promote epileptic hyperexcitability and accelerate kindling epileptogenesis (Ghanbarabadi & Sayyah, 2013; Pais et al., 2003). There might be two possible explanations for these paradoxical observations. On one hand, the role of quinine might depend on the mutual interaction between chemical and electric synapses and to what extent they were involved in different models of CNS diseases (Ghanbarabadi & Sayyah, 2013). On the other hand, it might be due to the off‐target effects of quinine at a higher dose (e.g., 400 μM), which was considered as a less likely explanation but could not be completely ruled out (Voss et al., 2009). Additionally, neither chronic stress nor quinine treatment produced an impact on the locomotor activity and anxious status of mice in OFT (Figure 3), indicating that chronic stress or quinine treatment made no difference to the excitability of central nervous system, which was consistent with other studies (Frisch et al., 2005; Su et al., 2018). In a recent study, neither 5 weeks nor 9 weeks of CUMS changed the locomotor activity of mice in OFT (Wang et al., 2021). As to quinine, similar results showed that knockout of Cx36 did not affect the locomotor activity of mice in OFT (Frisch et al., 2005; Steffensen et al., 2011). However, quinine might be able to improve the impaired locomotor activity in neuropsychiatric diseases (Nassiri‐Asl et al., 2009).

Inhibition of Cx36 by quinine could subsequently reduce the release of pro‐inflammatory cytokines like HMGB1, IL‐1β, and TNF‐α in the serum and hippocampus of mice exposed to CUMS, which was consistent with other studies. On the circumstances of injury, infection or stress, the innate immune system could be activated and induce inflammation afterwards to exert protective physiological effects (Vezzani et al., 2016). In most CNS diseases, a common underlying mechanism might be the inflammatory cascade reaction triggered by the release of inflammatory cytokines (Vezzani et al., 2016). Clinical evidence showed that inflammatory mediators were elevated in patients with depressive disorder, such as HMGB1, IL‐1β, TNF‐α, CRP, IL‐6, and so on (Leighton et al., 2018). In addition, animal studies also showed that in different models of depressive disorder, the inflammatory cytokines in the serum and CNS were increased significantly (Leighton et al., 2018; Miller et al., 2009). Furthermore, both clinical and experimental evidence indicated that administration of certain proinflammatory cytokines like IL‐1β, TNF‐α, or IFN‐α could induce depressive symptoms or depressive‐like behaviors, such as cognitive impairment, psychomotor retardation, reduced social, or exploratory behaviors (Dantzer et al., 2008; Eggermont et al., 2008; Friebe et al., 2010; Kim et al., 2016; Reichenberg et al., 2005). On the contrary, treated with anti‐inflammatory drugs or conditional knockout of inflammatory factors like HMGB1 could improve these symptoms (Coleman et al., 2018; Kappelmann et al., 2018; Musumeci et al., 2014; Tyring et al., 2006; Ye et al., 2019). There was also evidence that stress could enhance the activity of Cx36 HCs in neurons by direct or indirect overactivation of microglias, thus promoting the release of cytokines like TNF‐α and IL‐1β (Orellana et al., 2009; Sánchez et al., 2020). Treatment with quinine or GZA could in turn attenuate inflammatory symptoms through inhibition of certain proinflammatory cytokines, such as TNF‐α, IL‐1β, chemokine C‐C motif ligand (CCL) 3, and chemokine C‐X‐C motif ligand (CXCL) 8 (Grassin‐Delyle et al., 2019; Wang & Han et al., 2019). GZA might inhibit HMGB1 at first and subsequently reduce the levels of other inflammatory cytokines like TNF‐α and IL‐1β (Lian et al., 2017). However, the mechanisms underlying down‐regulation of inflammatory cytokines by quinine were complicated. In spite that quinine was a relatively specific inhibitor of Cx36, it could also bind to other proteins or ion channels to exert off‐target effects (Grassin‐Delyle et al., 2019; Maruyama et al., 1994). Therefore, more studies were needed to further invesitigate the mechanism.

In our study, an interesting finding drew our attention that HMGB1 and Cx36 might interact with each other mutually in the development of depressive disorder. Under CUMS, inhibition of HMGB1 by GZA could down‐regulate the expression of Cx36, while inhibition of Cx36 by quinine could in turn reduce the release of HMGB1 in the serum and hippocampus. Similar results were also found in other researches. Under nflammation, the release of HMGB1 by immune or nonimmune cells could be regulated by Cxs in the ultimate stage of nuclear translocation (Wang et al., 2020). The common mechanism might be the altered permission of Cx HCs in these processes mediated by the direct interaction with HMGB1 or its vectors. On the other hand, HMGB1 could also promote the release of other cytokines like TNF‐α and IL‐1β through regulation of Cx HCs in a study of osteocytes and osteoclasts (Davis et al., 2019). These results indicated that the possible mutual promotion of HMGB1 and Cx36 with each other in depressive disorder might promote the process, which were in need of further experiments.

Our results also showed that exposure to LPS could increase the excitability and influx of ions in hippocampal neurons, which was consistent with many studies. LPS was the important constituent in the outer membrane of Gram‐negative bacteria. It has been widely used in animal studies in vivo and vitro to explore the mechanisms underlying inflammatory CNS diseases (Laflamme et al., 2003; Rivest, 2003). Studies in vivo showed that intraperitoneal or intracerebroventricular injection of LPS could induce acute depressive‐like behaviors in rodents, which might be through the increase of proinflammatory mediators like HMGB1, IL‐1β, cyclooxygenase 2 (COX‐2), nitric oxide (NO), and prostaglandin (PG) (Leighton et al., 2018). Likewise, studies in vitro found that acute inflammation induced by LPS could promote the excitatory synaptic transmission and enhance neuronal excitability (Gao et al., 2014). One possibility was that the increased excitability was mediated by those immunogenic or inflammatory factors induced by LPS. For instance, IL‐1β could enhance the excitatory synaptic transmission through activation of N‐methyl‐D‐aspartate‐receptors (NMDARs), while TNF‐α could increase the frequency of spontaneous excitatory postsynaptic current (sEPSC) in hippocampal and spinal neurons (Gao et al., 2014). Our findings showed that LPS increased the amplitude and frequency of Aps, lowered the threshold and rheonobase currents, but did not affect the duration of Aps in hippocampal neurons. These were consistent with some studies, (Gomez et al., 2019) while others also found LPS did not obviously change the amplitude of Aps in hippocampal CA1pyramidal neurons (Gao et al., 2014). The possible explanation was that different kinds of inflammatory mediators induced by LPS might exert complex effects on ion channels, while our findings were the final comprehensive effects. For example, IL‐1β could activate voltage‐gated calcium channels and inhibit voltage‐gated sodium channels, while TNF‐α could up‐regulate voltage‐gated sodium channels (NAV1.3 and NAV1.8) and increase sodium influx. However, the underlying mechanisms how LPS enhanced neuronal excitability and synaptic transmission are not fully clarified. There was evidence that LPS could be recognized by TLR4 in microglia, thus inducing the release of proinflammatory cytokines (e.g., IL‐1β and TNF‐α), and then activating Cx HCs. The enhanced permeability of HCs could lead to the increase of Ca^2+^ influx and extracellular glutamate in hippocampal CA1 neurons. These findings indicated that activated microglia might change the activity of neurons through Cxs. In fact, augmented functions of Cxs between neurons and microglia were considered to be an important mechanism underlying the increased neuronal excitability in a variety of inflammatory CNS diseases (Medina‐Ceja et al., 2019).

In our study, we also observed that treatment with GZA or quinine could decrease the amplitude and frequency of APs, increase the threshold and rheonobase currents, and decrease inward currents, while application of GZA or quinine alone did not made any differences to the electrical characteristics of hippocample neurons. As mentioned above, GZA was an inactive analog of CBX that could not directly inhibit Cxs. Evidence showed that GZA itself did not affect the action potential, voltage‐gated Na^+^ current (*I*_Na_), or voltage‐gated K^+^ current (I_K_) evoked by depolarizing voltage steps (Elsen et al., 2008; Kimura et al., 1985). However, in the presence of other stimulators like glutamate or sustained depolarization (SD), GZA could diminish the inward Ca^2+^ current (Cherng et al., 2006; Kimura et al., 1985). As to quinine, evidence also showed that in normal artificial cerebrospinal fluid (ACSF), it did not produce influences on the resting membrane potential, input resistance (*R*_in_), AP threshold or amplitude of evoked population spikes (PS) in hippocampal CA1 pyramidal neurons (Bikson et al., 2002). The effects of quinine on the elecrical characteristics of neurons lied in the increase of the duration and decrease of the firing frequency of APs in the process of SD, but it produced no effects on normal resting membrane potentials (Bikson et al., 2002). Furthermore, quinine could inhibit extracellular transient potassium current and reduce the firing frequency in a voltage‐dependent manner (Cummings et al., 2008). There was also evidence that under normal membrane potential, quinine had no effect on the evoked current, but it could significantly inhibit the calcium influx during depolarization (Seemann et al., 2018). In spite of these observations, the in depth researches were still in need to further expound the underlying mechanisms.

## 5 CONCLUSIONS

**FIGURE 8:**
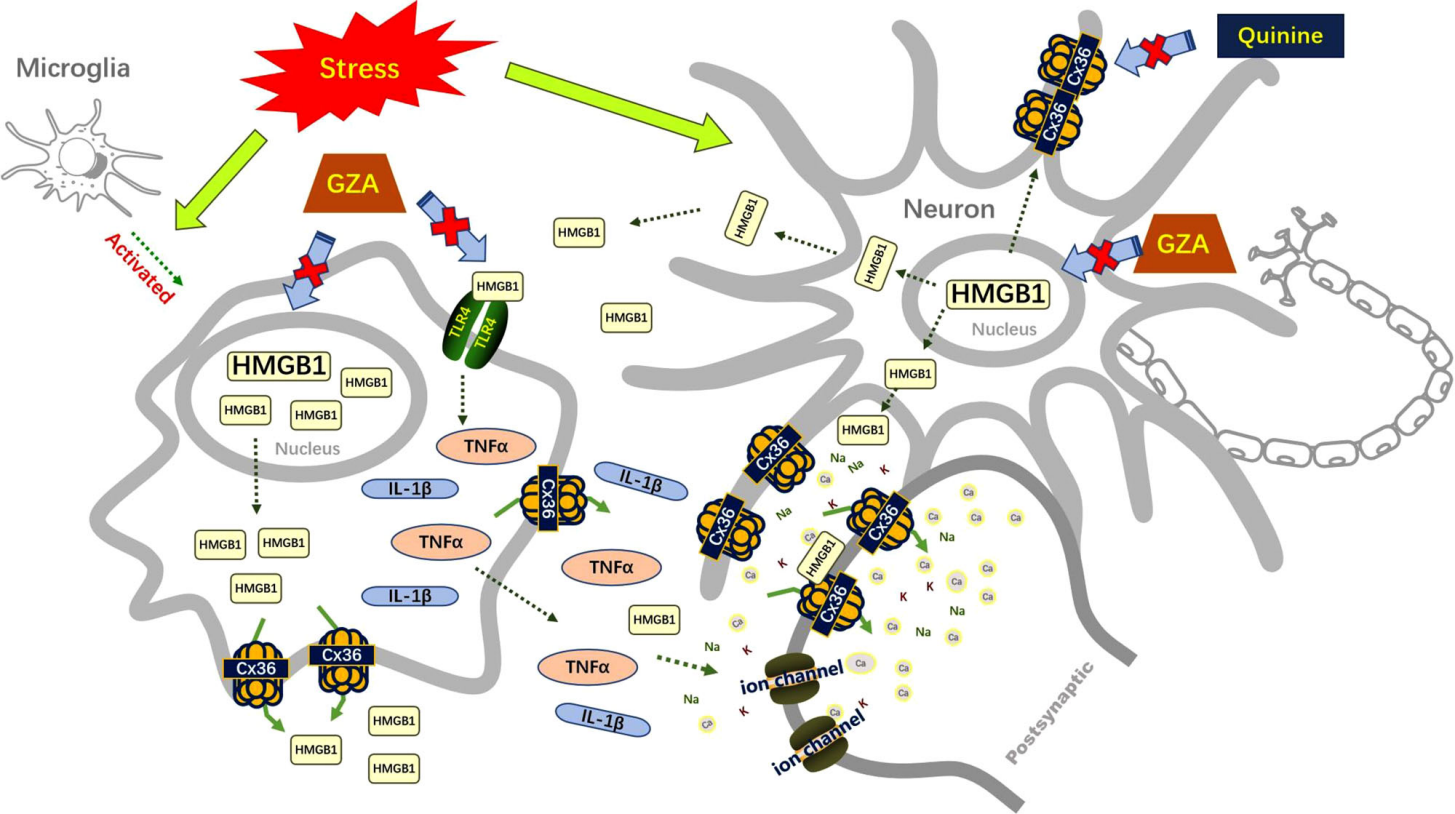
A working model to explain the therapeutic effects of Cx36 inhibition in HMGB1‐mediated depressive‐like behaviors induced by CUMS. Inhibition of Cx36 in the hippocampus could down‐regulate the increased HMGB1 induced by CUMS, as well as the downstream cytokines like TNF‐α and IL‐1β. Besides, inhibition of Cx36 and HMGB1 could also reduce the excitability and intracellular ion overload of hippocampal neurons. Cx36, Connexin36; HMGB1, high mobility group box 1; CUMS, chronic unpredictable mild stress; GZA, glycyrrhizinic acid; TLR4, toll‐like receptors 4; TNF‐α, tumor necrosis factor alpha; IL‐1β, interleukin‐1β

In summary, inhibition of Cx36 in hippocampal neurons might attenuate HMGB1 mediated depressive‐like behaviors induced by CUMS through down‐regulation of proinflammatory cytokines and reduction of the excitability and intracellular ion overload, thus playing an antidepressant role (Figure 8). Cx36 and HMGB1 might mutually promote each other in the development of depressive‐like behaviors so that inhibition of them might be of benefit to depressive disorder.

## CONFLICT OF INTEREST

The authors declare no conflict of interest.

## AUTHOR CONTRIBUTIONS

Qian Jiang and Chaoran Li conducted the experiments, drew the figures and wrote the manuscript. Wenfeng Zeng, Huijing Xu, Jiamei Li helped to perform the experiments. Ting Zhang analyzed the data. Yunxia Wang designed the study and collected the funds. Guanghui Deng and Yunxia Wang conceived the idea and revised the manuscript.

## ETHICAL APPROVAL

This study was approved by the experimental animal ethics committee of Second military Medical University.

### PEER REVIEW

The peer review history for this article is available at https://publons.com/publon/10.1002/brb3.2470

## Supporting information

Figure S1. GZA alleviated depressive‐like behaviors induced by CUMS. CUMS significantly decreased the sucrose preference (a) and extended the immobility duration of mice in TST(b) and FST (c), while treatment with GZA could markedly alleviate these symptoms (a–c). (*p < .05, **p < .01, *n* = 8–10 for each group). GZA: Glycyrrhizinic acid; CUMS: chronic unpredictable mild stress; TST: tail suspension test; FST: forced swimming test.
Figure S2. GZA decreased the release of pro‐inflammatory cytokines in the serum and hippocampus induced by CUMS. The serum levels of HMGB1 (a), TNF‐α (b) and IL‐1β (c) were significantly increased by CUMS, but decreased significantly after treatment with GZA (a–c). (*p < .05, **p < .01, *n* = 8–10 for each group) Similarly, the hippocampal levels of HMGB1 (D), TNF‐α (E) and IL‐1β (F) were also increased significantly by CUMS and decreased after quinine administration. (**p < .0l, ***p < .001, *n* = 8–10 for each group). GZA: Glycyrrhizinic acid; CUMS: chronic unpredictable mild stress; HMGB1: high mobility group box 1; TNF‐α: tumor necrosis factor alpha; IL‐1β: interleukin‐1β.

## Data Availability

All data included in this study are available upon request by contact with the corresponding author.

## References

[brb32470-bib-0001] Aguilar‐Valles, A. , Kim, J. , Jung, S. , Woodside, B. , & Luheshi, G. N . (2015). Role of brain transmigrating neutrophils in depression‐like behavior during systemic infection. Molecular Psychiatry, 20(3), 413–414. 10.1038/mp.2014.173 25644382

[brb32470-bib-0002] Allagnat, F. , Klee, P. , Cardozo, A. K. , Meda, P. , & Haefliger, J . (2013). Connexin36 contributes to INS‐1E cells survival through modulation of cytokine‐induced oxidative stress, ER stress and AMPK activity. Cell Death and Differentiation, 20(12), 1742–1752. 10.1038/cdd.2013.134 24096873 PMC3824597

[brb32470-bib-0003] Arundine, M. , & Tymianski, M . (2004). Molecular mechanisms of glutamate‐dependent neurodegeneration in ischemia and traumatic brain injury. Cellular and Molecular Life Sciences, 61(6), 657–668. 10.1007/s00018-003-3319-x 15052409 PMC11138528

[brb32470-bib-0004] Bailly, C. , & Vergoten, G . (2020). Glycyrrhizin: An alternative drug for the treatment of COVID‐19 infection and the associated respiratory syndrome? Pharmacology & Therapeutics, 214, 107618. 10.1016/j.pharmthera.2020.107618 32592716 PMC7311916

[brb32470-bib-0005] Beaudoin, G. M. 3rd , Lee, S. H. , Singh, D. , Yuan, Y. , Ng, Y. G. , Reichardt, L. F. , & Arikkath, J . (2012). Culturing pyramidal neurons from the early postnatal mouse hippocampus and cortex. Nature Protocols, 7(9), 1741–1754. 10.1038/nprot.2012.099 22936216

[brb32470-bib-0006] Belousov, A. B. , Fontes, J. D. , Freitas‐Andrade, M. , & Naus, C. C . (2017). Gap junctions and hemichannels: Communicating cell death in neurodevelopment and disease. BMC Cell Biology, 18(S1). 10.1186/s12860-016-0120-x PMC5267333 28124625

[brb32470-bib-0007] Beurel, E. , Toups, M. , & Nemeroff, C. B . (2020). The bidirectional relationship of depression and inflammation: Double trouble. Neuron, 107(2), 234–256. 10.1016/j.neuron.2020.06.002 32553197 PMC7381373

[brb32470-bib-0008] Bikson, M. , Id Bihi, R. , Vreugdenhil, M. , Köhling, R. , Fox, J. E. , & Jefferys, J. G . (2002). Quinine suppresses extracellular potassium transients and ictal epileptiform activity without decreasing neuronal excitability in vitro. Neuroscience, 115(1), 251–261. 10.1016/S0306-4522(02)00320-2 12401338

[brb32470-bib-0010] Cernackova, A. , Durackova, Z. , Trebaticka, J. , & Mravec, B . (2020). Neuroinflammation and depressive disorder: The role of the hypothalamus. Journal of Clinical Neuroscience, 75, 5–10. 10.1016/j.jocn.2020.03.005 32217047

[brb32470-bib-0011] Chen, J. , Wang, Z. Z. , Zuo, W. , Zhang, S. , Chu, S. F. , & Chen, N. H . (2016). Effects of chronic mild stress on behavioral and neurobiological parameters ‐ Role of glucocorticoid. Hormones and Behavior, 78, 150–159. 10.1016/j.yhbeh.2015.11.006 26592454

[brb32470-bib-0012] Chen, S. D. , Wang, Y. L. , Liang, S. F. , & Shaw, F. Z . (2016a). Rapid amygdala kindling causes motor seizure and comorbidity of anxiety‐ and depression‐like behaviors in rats. Frontiers in Behavioral Neuroscience, 10, 129. 10.3389/fnbeh.2016.00129 27445726 PMC4916743

[brb32470-bib-0013] Cherng, J. M. , Lin, H. J. , Hung, M. S. , Lin, Y. R. , Chan, M. H. , & Lin, J. C . (2006). Inhibition of nuclear factor κB is associated with neuroprotective effects of glycyrrhizic acid on glutamate‐induced excitotoxicity in primary neurons. European Journal of Pharmacology, 547(1‐3), 10–21. 10.1016/j.ejphar.2006.06.080 16952351

[brb32470-bib-0014] Coleman, L. G. , Zou, J. , Qin, L. , & Crews, F. T . (2018). HMGB1/IL‐1β complexes regulate neuroimmune responses in alcoholism. Brain, Behavior, and Immunity, 72, 61–77. 10.1016/j.bbi.2017.10.027 PMC5932292 29102800

[brb32470-bib-0015] Cryan, J. F. , Mombereau, C. , & Vassout, A . (2005). The tail suspension test as a model for assessing antidepressant activity: Review of pharmacological and genetic studies in mice. Neuroscience and Biobehavioral Reviews, 29(4‐5), 571–625. 10.1016/j.neubiorev.2005.03.009 15890404

[brb32470-bib-0016] Cryan, J. F. , & Holmes, A . (2005). The ascent of mouse: Advances in modelling human depression and anxiety. Nature Reviews Drug Discovery, 4(9), 775–790. 10.1038/nrd1825 16138108

[brb32470-bib-0017] Cryan, J. F. , & Slattery, D. A . (2007). Animal models of mood disorders: Recent developments. Current Opinion in Psychiatry, 20(1), 1–7. 10.1097/YCO.0b013e3280117733 17143074

[brb32470-bib-0018] Cummings, D. M. , Yamazaki, I. , Cepeda, C. , Paul, D. L. , & Levine, M. S . (2008). Neuronal coupling via connexin36 contributes to spontaneous synaptic currents of striatal medium‐sized spiny neurons. Journal of Neuroscience Research, 86(10), 2147–2158. 10.1002/jnr.21674 18381762

[brb32470-bib-0019] Dantzer, R. , O'Connor, J. C. , Freund, G. G. , Johnson, R. W. , & Kelley, K. W . (2008). From inflammation to sickness and depression: When the immune system subjugates the brain. Nature Reviews Neuroscience, 9(1), 46–56. 10.1038/nrn2297 18073775 PMC2919277

[brb32470-bib-0020] David, D. J. , Samuels, B. A. , Rainer, Q. , Wang, J. W. , Marsteller, D. , Mendez, I. , Drew, M. , Craig, D. A. , Guiard, B. P. , Guilloux, J. P. , Artymyshyn, R. P. , Gardier, A. M. , Gerald, C. , Antonijevic, I. A. , Leonardo, E. D. , & Hen, R . (2009). Neurogenesis‐dependent and ‐independent effects of fluoxetine in an animal model of anxiety/depression. Neuron, 62(4), 479–493. 10.1016/j.neuron.2009.04.017 19477151 PMC2759281

[brb32470-bib-0021] Davis, H. M. , Valdez, S. , Gomez, L. , Malicky, P. , White, F. A. , Subler, M. A. , Windle, J. J. , Bidwell, J. P. , Bruzzaniti, A. , & Plotkin, L. I . (2019). High mobility group box 1 protein regulates osteoclastogenesis through direct actions on osteocytes and osteoclasts in vitro. Journal of Cellular Biochemistry, 120(10), 16741–16749. 10.1002/jcb.28932 31106449 PMC6713577

[brb32470-bib-0022] Dobrenis, K. , Chang, H. Y. , Pina‐Benabou, M. H. , Woodroffe, A. , Lee, S. C. , Rozental, R. , Spray, D. C. , & Scemes, E . (2005). Human and mouse microglia express connexin36, and functional gap junctions are formed between rodent microglia and neurons. Journal of Neuroscience Research, 82(3), 306–315. 10.1002/jnr.20650 16211561 PMC2583240

[brb32470-bib-0023] Due, M. R. , Piekarz, A. D. , Wilson, N. , Feldman, P. , Ripsch, M. S. , Chavez, S. , Yin, H. , Khanna, R. , & White, F. A . (2012). Neuroexcitatory effects of morphine‐3‐glucuronide are dependent on Toll‐like receptor 4 signaling. Journal of Neuroinflammation, 9, 200. 10.1186/1742-2094-9-200 22898544 PMC3519737

[brb32470-bib-0024] Eggermont, A. M. , Suciu, S. , Santinami, M. , Testori, A. , Kruit, W. H. , Marsden, J. , Punt, C. J. , Salès, F. , Gore, M. , MacKie, R. , Kusic, Z. , Dummer, R. , Hauschild, A. , Musat, E. , Spatz, A. , Keilholz, U. , Melanoma Group E . (2008). Adjuvant therapy with pegylated interferon alfa‐2b versus observation alone in resected stage III melanoma: Final results of EORTC 18991, a randomised phase III trial. Lancet, 372(9633), 117–126. 10.1016/S0140-6736(08)61033-8 18620949

[brb32470-bib-0025] Elsen, F. P. , Shields, E. J. , Roe, M. T. , Vandam, R. J. , & Kelty, J. D . (2008). Carbenoxolone induced depression of rhythmogenesis in the pre‐Bötzinger Complex. BMC neuroscience, 9(1), 46. 10.1186/1471-2202-9-46 18500991 PMC2413244

[brb32470-bib-0026] Faridkia, Z. , Yaghmaei, P. , & Nassiri‐Asl, M . (2016). Protective effect of quinine on chemical kindling and passive avoidance test in rats. Iranian Red Crescent Medical Journal, 18(9), e25490. 10.5812/ircmj.25490 28144451 PMC5256102

[brb32470-bib-0027] Friebe, A. , Horn, M. , Schmidt, F. , Janssen, G. , Schmid‐Wendtner, M. H. , Volkenandt, M. , Hauschild, A. , Goldsmith, C. H. , & Schaefer, M . (2010). Dose‐dependent development of depressive symptoms during adjuvant interferon‐{alpha} treatment of patients with malignant melanoma. Psychosomatics, 51(6), 466–473. 10.1176/appi.psy.51.6.466 21051677

[brb32470-bib-0028] Frisch, C. , De Souza‐Silva, M. A. , Söhl, G. , Güldenagel, M. , Willecke, K. , Huston, J. P. , & Dere, E . (2005). Stimulus complexity dependent memory impairment and changes in motor performance after deletion of the neuronal gap junction protein connexin36 in mice. Behavioural Brain Research, 157(1), 177–185. 10.1016/j.bbr.2004.06.023 15617784

[brb32470-bib-0029] Fu, H. , Liu, L. , Tong, Y. , Li, Y. , Zhang, X. , Gao, X. , Yong, J. , Zhao, J. , Xiao, D. , Wen, K. , & Wang, H . (2019). The antidepressant effects of hesperidin on chronic unpredictable mild stress‐induced mice. European Journal of Pharmacology, 853, 236–246. 10.1016/j.ejphar.2019.03.035 30928632

[brb32470-bib-0030] Gajda, Z. , Szupera, Z. , Blazsó, G. , & Szente, M . (2005). Quinine, a blocker of neuronal Cx36 channels, suppresses seizure activity in rat neocortex in vivo. Epilepsia (Copenhagen), 46(10), 1581–1591. 10.1111/j.1528-1167.2005.00254.x 16190928

[brb32470-bib-0031] Gao, F. , Liu, Z. , Ren, W. , & Jiang, W . (2014). Acute lipopolysaccharide exposure facilitates epileptiform activity via enhanced excitatory synaptic transmission and neuronal excitability in vitro. Neuropsychiatric Disease and Treatment, 10, 1489–1495. 10.2147/NDT.S65695 25170268 PMC4144925

[brb32470-bib-0032] Gardner, A. , Jukkola, P. , & Gu, C . (2012). Myelination of rodent hippocampal neurons in culture. Nature Protocols, 7(10), 1774–1782. 10.1038/nprot.2012.100 22955693 PMC3536533

[brb32470-bib-0033] Garrett, F. G. , & Durham, P. L . (2008). Differential expression of connexins in trigeminal ganglion neurons and satellite glial cells in response to chronic or acute joint inflammation. Neuron Glia Biology, 4(4), 295–306. 10.1017/S1740925X09990093 19674505 PMC3138122

[brb32470-bib-0034] GBD 2017 Disease and Injury Incidence and Prevalence Collaborators . (2018). Global, regional, and national incidence, prevalence, and years lived with disability for 354 diseases and injuries for 195 countries and territories, 1990–2017: A systematic analysis for the Global Burden of Disease Study 2017. Lancet, 392(10159), 1789–1858. 10.1016/S0140-6736(18)32279-7 30496104 PMC6227754

[brb32470-bib-0035] Ghanbarabadi, J. K. , & Sayyah, M . (2013). Blocking of rat hippocampal Cx36 by quinine accelerates kindling epileptogenesis. EXCLI Journal, 12, 251–259 26417230 PMC4552103

[brb32470-bib-0036] Gomez, C. D. , Read, J. , Acharjee, S. , & Pittman, Q. J . (2019). Early life inflammation increases CA1 pyramidal neuron excitability in a sex and age dependent manner through a chloride homeostasis disruption. The Journal of Neuroscience, 39(37), 7244–7259. 10.1523/JNEUROSCI.2973-18.2019 31308096 PMC6759023

[brb32470-bib-0037] Grassin‐Delyle, S. , Salvator, H. , Mantov, N. , Abrial, C. , Brollo, M. , Faisy, C. , Naline, E. , Couderc, L. J. , & Devillier, P . (2019). Bitter taste receptors (TAS2Rs) in human lung macrophages: Receptor expression and inhibitory effects of TAS2R agonists. Frontiers in Physiology, 10, 1267. 10.3389/fphys.2019.01267 31632299 PMC6783802

[brb32470-bib-0038] Hamzei‐Sichani, F. , Davidson, K. G. , Yasumura, T. , Janssen, W. G. , Wearne, S. L. , Hof, P. R. , Traub, R. D. , Gutiérrez, R. , Ottersen, O. P. , & Rash, J. E . (2012). Mixed electrical–chemical synapses in adult rat hippocampus are primarily glutamatergic and coupled by connexin‐36. Frontiers in Neuroanatomy, 6. 10.3389/fnana.2012.00013 PMC3351785 22615687

[brb32470-bib-0039] Hanstein, R. , Trotter, J. , Behl, C. , & Clement, A. B . (2009). Increased connexin 43 expression as a potential mediator of the neuroprotective activity of the corticotropin‐releasing hormone. Molecular Endocrinology, 23(9), 1479–1493. 10.1210/me.2009-0022 19460861 PMC2737551

[brb32470-bib-0040] Huang, Y. , Wang, Y. , Wang, H. , Liu, Z. , Yu, X. , Yan, J. , Yu, Y. , Kou, C. , Xu, X. , Lu, J. , Wang, Z. , He, S. , Xu, Y. , He, Y. , Li, T. , Guo, W. , Tian, H. , Xu, G. , Xu, X ., … Wu, Y . (2019). Prevalence of mental disorders in China: A cross‐sectional epidemiological study. The Lancet Psychiatry, 6(3), 211–224. 10.1016/S2215-0366(18)30511-X 30792114

[brb32470-bib-0041] Jeanson, T. , Pondaven, A. , Ezan, P. , Mouthon, F. , Charvériat, M. , & Giaume, C . (2016). Antidepressants impact connexin 43 channel functions in astrocytes. Frontiers in Cellular Neuroscience, 9. 10.3389/fncel.2015.00495 PMC4703821 26778961

[brb32470-bib-0042] Kaech, S. , & Banker, G . (2006). Culturing hippocampal neurons. Nature Protocols, 1(5), 2406–2415. 10.1038/nprot.2006.356 17406484

[brb32470-bib-0043] Kappelmann, N. , Lewis, G. , Dantzer, R. , Jones, P. B. , & Khandaker, G. M . (2018). Antidepressant activity of anti‐cytokine treatment: A systematic review and meta‐analysis of clinical trials of chronic inflammatory conditions. Molecular Psychiatry, 23(2), 335–343. 10.1038/mp.2016.167 27752078 PMC5794896

[brb32470-bib-0044] Kim, Y. K. , Na, K. S. , Myint, A. M. , & Leonard, B. E . (2016). The role of pro‐inflammatory cytokines in neuroinflammation, neurogenesis and the neuroendocrine system in major depression. Progress in Neuro‐Psychopharmacology & Biological Psychiatry, 64, 277–284. 10.1016/j.pnpbp.2015.06.008 26111720

[brb32470-bib-0045] Kimura, M. , Kimura, I. , & Nojima, H . (1985). Depolarizing neuromuscular blocking action induced by electropharmacological coupling in the combined effect of paeoniflorin and glycyrrhizin. Japanese Journal of Pharmacology, 37(4), 395–399. 10.1254/jjp.37.395 4010089

[brb32470-bib-0046] Köster‐Patzlaff, C. , Hosseini, S. M. , & Reuss, B . (2009). Loss of connexin36 in rat hippocampus and cerebellar cortex in persistent Borna disease virus infection. Journal of Chemical Neuroanatomy, 37(2), 118–127. 10.1016/j.jchemneu.2008.10.004 19038327

[brb32470-bib-0047] Laflamme, N. , Echchannaoui, H. , Landmann, R. , & Rivest, S . (2003). Cooperation between toll‐like receptor 2 and 4 in the brain of mice challenged with cell wall components derived from gram‐negative and gram‐positive bacteria. European Journal of Immunology, 33(4), 1127–1138. 10.1002/eji.200323821 12672079

[brb32470-bib-0048] Leighton, S. P. , Nerurkar, L. , Krishnadas, R. , Johnman, C. , Graham, G. J. , & Cavanagh, J . (2018). Chemokines in depression in health and in inflammatory illness: A systematic review and meta‐analysis. Molecular Psychiatry, 23(1), 48–58. 10.1038/mp.2017.205 29133955 PMC5754468

[brb32470-bib-0049] Leng, L. , Zhuang, K. , Liu, Z. , Huang, C. , Gao, Y. , Chen, G. , Lin, H. , Hu, Y. , Wu, D. , Shi, M. , Xie, W. , Sun, H. , Shao, Z. , Li, H. , Zhang, K. , Mo, W. , Huang, T. Y. , Xue, M. , Yuan, Z. , … Zhang, J. (2018). Menin deficiency leads to depressive‐like behaviors in mice by modulating astrocyte‐mediated neuroinflammation. Neuron, 100(3), 551–563. 10.1016/j.neuron.2018.08.031 30220511

[brb32470-bib-0051] Lian, Y. J. , Gong, H. , Wu, T. Y. , Su, W. J. , Zhang, Y. , Yang, Y. Y. , Peng, W. , Zhang, T. , Zhou, J. R. , Jiang, C. L. , & Wang, Y. X . (2017). Ds‐HMGB1 and fr‐HMGB induce depressive behavior through neuroinflammation in contrast to nonoxid‐HMGB1. Brain, Behavior, and Immunity, 59, 322–332. 10.1016/j.bbi.2016.09.017 27647532

[brb32470-bib-0052] Liu, C. H. , Zhang, G. Z. , Li, B. , Li, M. , Woelfer, M. , Walter, M. , & Wang, L . (2019). Role of inflammation in depression relapse. Journal of Neuroinflammation, 16(1). 10.1186/s12974-019-1475-7 PMC6472093 30995920

[brb32470-bib-0053] Malhi, G. S. , & Mann, J. J . (2018). Depression. Lancet, 392(10161), 2299–2312. 10.1016/S0140-6736(18)31948-2 30396512

[brb32470-bib-0054] Maruyama, N. , Kakuta, Y. , Yamauchi, K. , Ohkawara, Y. , Aizawa, T. , Ohrui, T. , Nara, M. , Oshiro, T. , Ohno, I. , & Tamura, G . (1994). Quinine inhibits production of tumor necrosis factor‐alpha from human alveolar macrophages. American Journal of Respiratory Cell and Molecular biology, 10(5), 514–520. 10.1165/ajrcmb.10.5.8179913 8179913

[brb32470-bib-0055] McCracken, C. B. , Hamby, S. M. , Patel, K. M. , Morgan, D. , Vrana, K. E. , & Roberts, D. C . (2005). Extended cocaine self‐administration and deprivation produces region‐specific and time‐dependent changes in connexin36 expression in rat brain. Synapse, 58(3), 141–150. 10.1002/syn.20194 16138316

[brb32470-bib-0056] Medina‐Ceja, L. , Salazar‐Sanchez, J. C. , Ortega‐Ibarra, J. , & Morales‐Villagran, A . (2019). Connexins‐based hemichannels/channels and their relationship with inflammation, seizures and epilepsy. International Journal of Molecular Sciences, 20(23). 10.3390/ijms20235976 PMC6929063 31783599

[brb32470-bib-0057] Mesnil, M. , Defamie, N. , Naus, C. , & Sarrouilhe, D . (2021). Brain disorders and chemical pollutants: A gap junction link? Biomolecules, 11(1), 51. 10.3390/biom11010051 PMC7824109 33396565

[brb32470-bib-0058] Miller, A. H. , Maletic, V. , & Raison, C. L . (2009). Inflammation and its discontents: The role of cytokines in the pathophysiology of major depression. Biological Psychiatry, 65(9), 732–741. 10.1016/j.biopsych.2008.11.029 19150053 PMC2680424

[brb32470-bib-0059] Mitrou, N. , Braam, B. , & Cupples, W. A . (2016). A gap junction inhibitor, carbenoxolone, induces spatiotemporal dispersion of renal cortical perfusion and impairs autoregulation. American Journal of Physiology‐Heart and Circulatory Physiology, 311(3), H582‐H591. 10.1152/ajpheart.00941.2015 27371687

[brb32470-bib-0060] Musumeci, D. , Roviello, G. N. , & Montesarchio, D . (2014). An overview on HMGB1 inhibitors as potential therapeutic agents in HMGB1‐related pathologies. Pharmacology & Therapeutics, 141(3), 347–357. 10.1016/j.pharmthera.2013.11.001 24220159

[brb32470-bib-0061] Nagy, J. I. , & Rash, J. E . (2017). Cx36, Cx43 and Cx45 in mouse and rat cerebellar cortex: Species‐specific expression, compensation in Cx36 null mice and co‐localization in neurons vs. glia. European Journal of Neuroscience, 46(2), 1790–1804. 10.1111/ejn.13614 28561933

[brb32470-bib-0062] Nassiri‐Asl, M. , Zamansoltani, F. , & Torabinejad, B . (2009). Antiepileptic effects of quinine in the pentylenetetrazole model of seizure. Seizure: The Journal of the British Epilepsy Association, 18(2), 129–132. 10.1016/j.seizure.2008.08.002 18786839

[brb32470-bib-0063] Nassiri‐Asl, M. , Zamansoltani, F. , & Zangivand, A. A . (2008). The inhibitory effect of trimethylamine on the anticonvulsant activities of quinine in the pentylenetetrazole model in rats. Progress in Neuro‐Psychopharmacology & Biological Psychiatry, 32(6), 1496–1500. 10.1016/j.pnpbp.2008.05.007 18556104

[brb32470-bib-0064] Orellana, J. A. , Moraga‐Amaro, R. , Díaz‐Galarce, R. , Rojas, S. , Maturana, C. J. , Stehberg, J. , & Sáez, J. C . (2015). Restraint stress increases hemichannel activity in hippocampal glial cells and neurons. Front Cell Neurosci, 9, 102. 10.3389/fncel.2015.00102 25883550 PMC4382970

[brb32470-bib-0065] Orellana, J. A. , Sáez, P. J. , Shoji, K. F. , Schalper, K. A. , Palacios‐Prado, N. , Velarde, V. , Giaume, C. , Bennett, M. V. , & Sáez, J. C . (2009). Modulation of brain hemichannels and gap junction channels by pro‐inflammatory agents and their possible role in neurodegeneration. Antioxidants & Redox Signaling, 11(2), 369–399. 10.1089/ars.2008.2130 18816186 PMC2713807

[brb32470-bib-0066] Otte, C. , Gold, S. M. , Penninx, B. W. , Pariante, C. M. , Etkin, A. , Fava, M. , Mohr, D. C. , & Schatzberg, A. F . (2016). Major depressive disorder. Nature Reviews Disease Primers, 2(1), 16065. 10.1038/nrdp.2016.65 27629598

[brb32470-bib-0067] Pais, I. , Hormuzdi, S. G. , Monyer, H. , Traub, R. D. , Wood, I. C. , Buhl, E. H. , Whittington, M. A. , & LeBeau, F. E . (2003). Sharp wave‐like activity in the hippocampus in vitro in mice lacking the gap junction protein connexin 36. Journal of Neurophysiology, 89(4), 2046–2054. 10.1152/jn.00549.2002 12686578

[brb32470-bib-0068] Patrone, L. G. , Bicego, K. C. , Hartzler, L. K. , Putnam, R. W. , & Gargaglioni, L. H . (2014). Cardiorespiratory effects of gap junction blockade in the locus coeruleus in unanesthetized adult rats. Respiratory Physiology & Neurobiology, 190, 86–95. 10.1016/j.resp.2013.09.001 24035835

[brb32470-bib-0069] Quesseveur, G. , Portal, B. , Basile, J. A. , Ezan, P. , Mathou, A. , Halley, H. , Leloup, C. , Fioramonti, X. , Déglon, N. , Giaume, C. , Rampon, C. , & Guiard, B. P . (2015). Attenuated levels of hippocampal connexin 43 and its phosphorylation correlate with antidepressant‐ and anxiolytic‐like activities in mice. Frontiers in Cellular Neuroscience, 9, 490. 10.3389/fncel.2015.00490 26733815 PMC4686612

[brb32470-bib-0070] Rainer, Q. , Nguyen, H. T. , Quesseveur, G. , Gardier, A. M. , David, D. J. , & Guiard, B. P . (2012). Functional status of somatodendritic serotonin 1A autoreceptor after long‐term treatment with fluoxetine in a mouse model of anxiety/depression based on repeated corticosterone administration. Molecular Pharmacology, 81(2), 106–112. 10.1124/mol.111.075796 22031471

[brb32470-bib-0071] Reed, G. M. , First, M. B. , Kogan, C. S. , Hyman, S. E. , Gureje, O. , Gaebel, W. , Maj, M. , Stein, D. J. , Maercker, A. , Tyrer, P. , Claudino, A. , Garralda, E. , Salvador‐Carulla, L. , Ray, R. , Saunders, J. B. , Dua, T. , Poznyak, V. , Medina‐Mora, M. E. , Pike, K. M. , … Saxena S . (2019). Innovations and changes in the ICD‐11 classification of mental, behavioural and neurodevelopmental disorders. World Psychiatry, 18(1), 3–19. 10.1002/wps.20611 30600616 PMC6313247

[brb32470-bib-0072] Reichenberg, A. , Gorman, J. M. , & Dieterich, D. T . (2005). Interferon‐induced depression and cognitive impairment in hepatitis C virus patients: A 72 week prospective study. Aids, 19 (Suppl 3), S174‐S178. 10.1097/01.aids.0000192087.64432.ae 16251815

[brb32470-bib-0073] Ren, Q. , Wang, Z. , Chu, S. , Xia, C. , & Chen, N . (2018). Gap junction channels as potential targets for the treatment of major depressive disorder. Psychopharmacology, 235(1), 1–12. 10.1007/s00213-017-4782-7 29178009

[brb32470-bib-0074] Rivest, S . (2003). Molecular insights on the cerebral innate immune system. Brain, Behavior, and Immunity, 17(1), 13–19. 10.1016/S0889-1591(02)00055-7 12615045

[brb32470-bib-0075] Sánchez, O. F. , Rodríguez, A. V. , Velasco‐España, J. M. , Murillo, L. C. , Sutachan, J. J. , & Albarracin, S. L . (2020). Role of connexins 30, 36, and 43 in brain tumors, neurodegenerative diseases, and neuroprotection. Cells, 9(4), 846. 10.3390/cells9040846 PMC7226843 32244528

[brb32470-bib-0076] Sarrouilhe, D. , Dejean, C. , & Mesnil, M . (2014). Involvement of gap junction channels in the pathophysiology of migraine with aura. Frontiers in Physiology, 5. 10.3389/fphys.2014.00078 PMC3933780 24611055

[brb32470-bib-0077] Seemann, N. , Welling, A. , & Rustenbeck, I . (2018). The inhibitor of connexin Cx36 channels, mefloquine, inhibits voltage‐dependent Ca(2+) channels and insulin secretion. Molecular and Cellular Endocrinology, 472, 97–106. 10.1016/j.mce.2017.11.024 29208420

[brb32470-bib-0078] Srinivas, M. , Hopperstad, M. G. , & Spray, D. C . (2001). Quinine blocks specific gap junction channel subtypes. Proceedings of the National Academy of Sciences ‐ PNAS, 98(19), 10942–10947. 10.1073/pnas.191206198 PMC58578 11535816

[brb32470-bib-0079] Steffensen, S. C. , Bradley, K. D. , Hansen, D. M. , Wilcox, J. D. , Wilcox, R. S. , Allison, D. W. , Merrill, C. B. , & Edwards, J. G . (2011). The role of connexin‐36 gap junctions in alcohol intoxication and consumption. Synapse, 65(8), 695–707. 10.1002/syn.20885 21638336 PMC3051038

[brb32470-bib-0080] Su, W. J. , Zhang, T. , Jiang, C. L. , & Wang, W . (2018). Clemastine alleviates depressive‐like behavior through reversing the imbalance of microglia‐related pro‐inflammatory state in mouse hippocampus. Frontiers in Cellular Neuroscience, 12, 412. 10.3389/fncel.2018.00412 30483062 PMC6243034

[brb32470-bib-0081] Takeuchi, H. , & Suzumura, A . (2014). Gap junctions and hemichannels composed of connexins: Potential therapeutic targets for neurodegenerative diseases. Frontiers in Cellular Neuroscience, 8, 189. 10.3389/fncel.2014.00189 25228858 PMC4151093

[brb32470-bib-0082] Tyring, S. , Gottlieb, A. , Papp, K. , Gordon, K. , Leonardi, C. , Wang, A. , Lalla, D. , Woolley, M. , Jahreis, A. , Zitnik, R. , Cella, D. , & Krishnan, R . (2006). Etanercept and clinical outcomes, fatigue, and depression in psoriasis: Double‐blind placebo‐controlled randomised phase III trial. Lancet, 367(9504), 29–35. 10.1016/S0140-6736(05)67763-X 16399150

[brb32470-bib-0083] Vezzani, A. , Fujinami, R. S. , White, H. S. , Preux, P. M. , Blümcke, I. , Sander, J. W. , & Löscher, W . (2016). Infections, inflammation and epilepsy. Acta Neuropathologica, 131(2), 211–234. 10.1007/s00401-015-1481-5 26423537 PMC4867498

[brb32470-bib-0084] Voss, L. J. , Jacobson, G. , Sleigh, J. W. , Steyn‐Ross, A. , & Steyn‐Ross, M . (2009). Excitatory effects of gap junction blockers on cerebral cortex seizure‐like activity in rats and mice. Epilepsia, 50(8), 1971–1978. 10.1111/j.1528-1167.2009.02087.x 19486358

[brb32470-bib-0085] Walker, A. K. , Wing, E. E. , Banks, W. A. , & Dantzer, R . (2019). Leucine competes with kynurenine for blood‐to‐brain transport and prevents lipopolysaccharide‐induced depression‐like behavior in mice. Molecular Psychiatry, 24(10), 1523–1532. 10.1038/s41380-018-0076-7 29988087 PMC6326900

[brb32470-bib-0086] Wang, B. , Huang, X. , Pan, X. , Zhang, T. , Hou, C. , Su, W. J. , Liu, L. L. , Li, J. M. , & Wang, Y. X . (2020). Minocycline prevents the depressive‐like behavior through inhibiting the release of HMGB1 from microglia and neurons. Brain, Behavior, and Immunity, 88, 132–143. 10.1016/j.bbi.2020.06.019 32553784

[brb32470-bib-0087] Wang, B. , Lian, Y. J. , Dong, X. , Peng, W. , Liu, L. L. , Su, W. J. , Gong, H. , Zhang, T. , Jiang, C. L. , Li, J. S. , & Wang, Y. X . (2018). Glycyrrhizic acid ameliorates the kynurenine pathway in association with its antidepressant effect. Behavioural Brain Research, 353, 250–257. 10.1016/j.bbr.2018.01.024 29366745

[brb32470-bib-0088] Wang, B. , Lian, Y. J. , Su, W. J. , Liu, L. L. , Li, J. M. , Jiang, C. L. , & Wang, Y. X . (2019). FrHMGB1 and dsHMGB1 activate the kynurenine pathway via different mechanisms in association with depressivelike behavior. Molecular Medicine Reports, 20(1), 359–367. 10.3892/mmr.2019.10225 31115516 PMC6580048

[brb32470-bib-0089] Wang, B. , Lian, Y. J. , Su, W. J. , Peng, W. , Dong, X. , Liu, L. L. , Gong, H. , Zhang, T. , Jiang, C. L. , & Wang, Y. X . (2018). HMGB1 mediates depressive behavior induced by chronic stress through activating the kynurenine pathway. Brain, Behavior, and Immunity, 72, 51–60. 10.1016/j.bbi.2017.11.017 29195782

[brb32470-bib-0090] Wang, D. , Wang, W. , Jiang, S. , Ma, H. , Lian, H. , Meng, F. , Liu, J. , Cui, M. , You, J. , Liu, C. , Zhao, D. , Hu, F. , Liu, D. , & Li, C . (2021). Regulation of depression‐related behaviors by GABAergic neurons in the lateral septum through periaqueductal gray neuronal projections. Journal of Psychiatric Research, 137, 202–214. 10.1016/j.jpsychires.2021.02.043 33691232

[brb32470-bib-0091] Wang, H. , Bloom, O. , Zhang, M. , Vishnubhakat, J. M. , Ombrellino, M. , Che, J. , Frazier, A. , Yang, H. , Ivanova, S. , Borovikova, L. , Manogue, K. R. , Faist, E. , Abraham, E. , Andersson, J. , Andersson, U. , Molina, P. E. , Abumrad, N. N. , Sama, A. , & Tracey, K. J . (1999). HMG‐1 as a late mediator of endotoxin lethality in mice. Science, 285(5425), 248–251. 10.1126/science.285.5425.248 10398600

[brb32470-bib-0092] Wang, R. , Yang, Y. , Xiao, M. , Guo, B. , Liu, W. , & Wang, H . (2019). Neonatal inhibition of connexin 36 ameliorates fetal brain injury induced by maternal noninfectious fever in mice. Developmental Neuroscience, 41(1‐2), 94–101. 10.1159/000499735 31112950

[brb32470-bib-0093] Wang, S. , Sun, Y. , Bai, Y. , Zhou, N. , Chen, N. , Miller, E. J. , Zhang, Y. , & Li, W . (2020). Contribution of connexin hemichannels to the pathogenesis of acute lung injury. Mediators of Inflammation, 2020, 1–10. 10.1155/2020/8094347 PMC7688369 33293898

[brb32470-bib-0094] Wang, Y. L. , Wu, H. R. , Zhang, S. S. , Xiao, H. L. , Yu, J. , Ma, Y. Y. , Zhang, Y. D. , & Liu, Q . (2021). Catalpol ameliorates depressive‐like behaviors in CUMS mice via oxidative stress‐mediated NLRP3 inflammasome and neuroinflammation. Translational Psychiatry, 11(1), 353. 10.1038/s41398-021-01468-7 34103482 PMC8187638

[brb32470-bib-0095] Wang, Z. , Han, N. , Zhao, K. , Li, Y. , Chi, Y. , & Wang, B . (2019). Protective effects of pyrroloquinoline quinine against oxidative stress‐induced cellular senescence and inflammation in human renal tubular epithelial cells via Keap1/Nrf2 signaling pathway. International Immunopharmacology, 72, 445–453. 10.1016/j.intimp.2019.04.040 31035086

[brb32470-bib-0096] Wu, T. Y. , Liu, L. , Zhang, W. , Zhang, Y. , Liu, Y. Z. , Shen, X. L. , Gong, H. , Yang, Y. Y. , Bi, X. Y. , Jiang, C. L. , & Wang, Y. X . (2015). High‐mobility group box‐1 was released actively and involved in LPS induced depressive‐like behavior. Journal of Psychiatric Research, 64, 99–106. 10.1016/j.jpsychires.2015.02.016 25795092

[brb32470-bib-0097] Xie, J. , Bi, B. , Qin, Y. , Dong, W. , Zhong, J. , Li, M. , Cheng, Y. , Xu, J. , & Wang, H . (2021). Inhibition of phosphodiesterase‐4 suppresses HMGB1/RAGE signaling pathway and NLRP3 inflammasome activation in mice exposed to chronic unpredictable mild stress. Brain, Behavior, and Immunity, 92, 67–77. 10.1016/j.bbi.2020.11.029 33221489

[brb32470-bib-0098] Yang, F. , Zhu, W. , Cai, X. , Zhang, W. , Yu, Z. , Li, X. , Zhang, J. , Cai, M. , Xiang, J. , & Cai, D . (2020). Minocycline alleviates NLRP3 inflammasome‐dependent pyroptosis in monosodium glutamate‐induced depressive rats. Biochemical and Biophysical Research Communications, 526(3), 553–559. 10.1016/j.bbrc.2020.02.149 32245616

[brb32470-bib-0099] Yang, P. , Gao, Z. , Zhang, H. , Fang, Z. , Wu, C. , Xu, H. , & Huang, Q. J . (2015). Changes in proinflammatory cytokines and white matter in chronically stressed rats. Neuropsychiatric Disease and Treatment, 11, 597–607. 10.2147/NDT.S78131 25834438 PMC4358419

[brb32470-bib-0100] Ye, Y. , Zeng, Z. , Jin, T. , Zhang, H. , Xiong, X. , & Gu, L . (2019). The role of high mobility group box 1 in ischemic stroke. Frontiers in Cellular Neuroscience, 13, 127. 10.3389/fncel.2019.00127 31001089 PMC6454008

[brb32470-bib-0101] Zolfaghari, F. S. , Pirri, F. , Gauvin, E. , Peeri, M. , & Amiri, S . (2021). Exercise and fluoxetine treatment during adolescence protect against early life stress‐induced behavioral abnormalities in adult rats. Pharmacology, Biochemistry and Behavior, 205, 173190. 10.1016/j.pbb.2021.173190 33865889

