# Supplementary figures and images for: Inhibition of Connexin 36 attenuates HMGB1‐mediated depressive‐like behaviors induced by chronic unpredictable mild stress
Source: Brain Behav. 2022 Jan 28;12(2):e2470. doi: 10.1002/brb3.2470 (PMC8865165; doi:10.1002/brb3.2470)

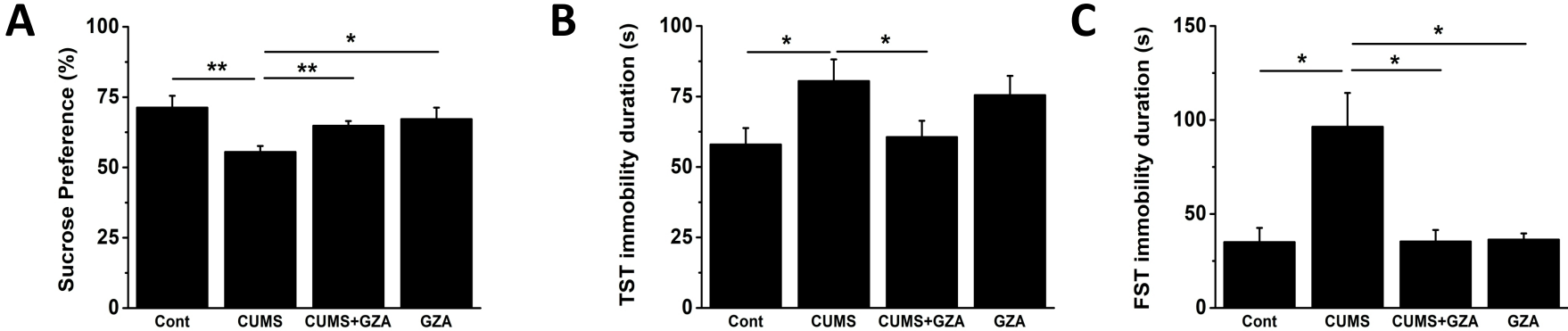

Fig. S1

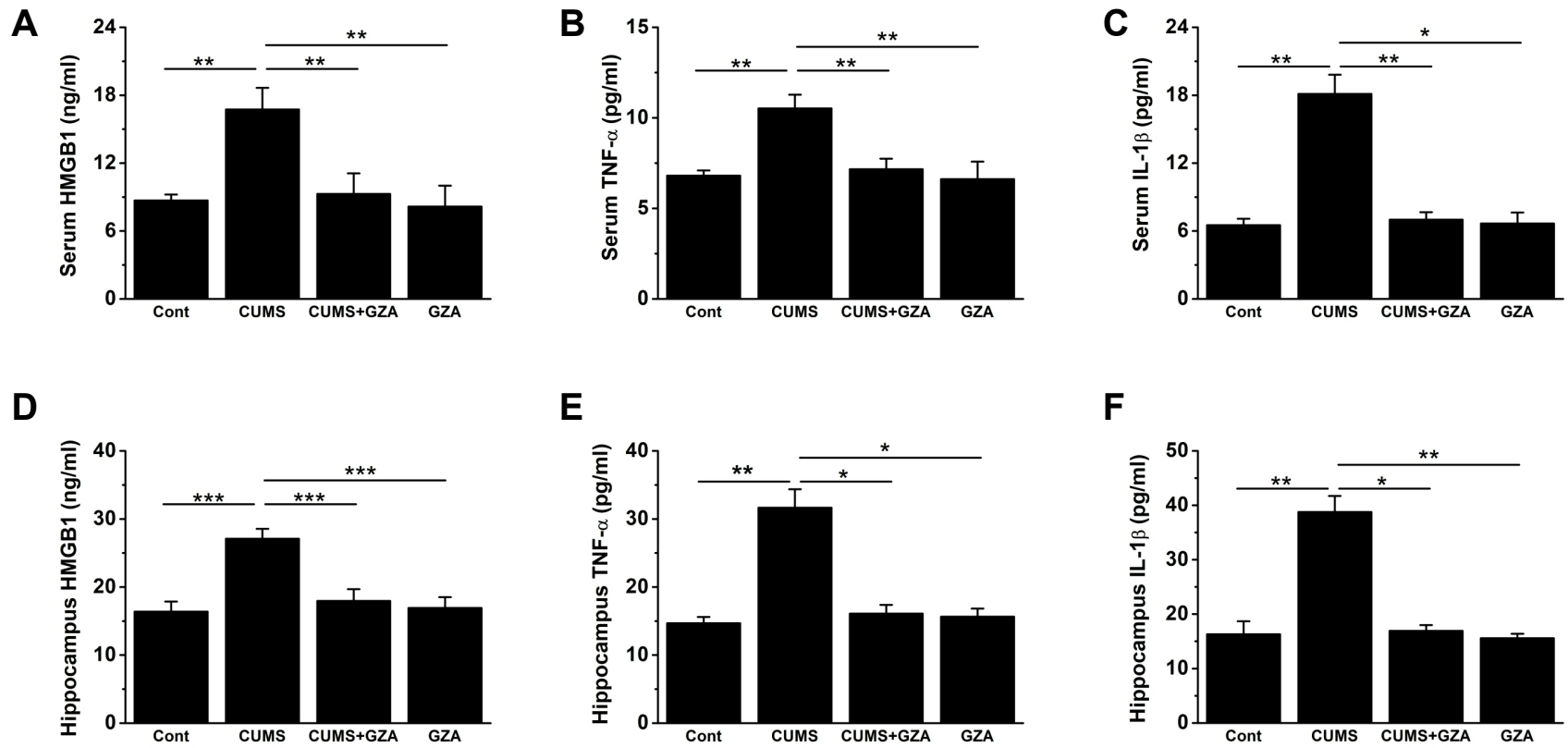

Fig. S2

Supplement: Supplementary file 1 — Figure S1. GZA alleviated depressive‐like behaviors induced by CUMS. CUMS significantly decreased the sucrose preference (a) and extended the immobility duration of mice in TST(b) and FST (c), while treatment with GZA could markedly alleviate these symptoms (a–c). (*p < .05, **p < .01, n = 8–10 for each group). GZA: Glycyrrhizinic acid; CUMS: chronic unpredictable mild stress; TST: tail suspension test; FST: forced swimming test. Figure S2. GZA decreased the release of pro‐inflammatory cytokines in the serum and hippocampus induced by CUMS. The serum levels of HMGB1 (a), TNF‐α (b) and IL‐1β (c) were significantly increased by CUMS, but decreased significantly after treatment with GZA (a–c). (*p < .05, **p < .01, n = 8–10 for each group) Similarly, the hippocampal levels of HMGB1 (D), TNF‐α (E) and IL‐1β (F) were also increased significantly by CUMS and decreased after quinine administration. (**p < .0l, ***p < .001, n = 8–10 for each group). GZA: Glycyrrhizinic acid; CUMS: chronic unpredictable mild stress; HMGB1: high mobility group box 1; TNF‐α: tumor necrosis factor alpha; IL‐1β: interleukin‐1β. [file BRB3-12-e2470-s001.pdf]
